# mRNA structure regulates protein expression through changes in functional half-life

**DOI:** 10.1073/pnas.1908052116

**Published:** 2019-11-11

**Authors:** David M. Mauger, B. Joseph Cabral, Vladimir Presnyak, Stephen V. Su, David W. Reid, Brooke Goodman, Kristian Link, Nikhil Khatwani, John Reynders, Melissa J. Moore, Iain J. McFadyen

**Affiliations:** ^a^Platform Research, Moderna, Inc., Cambridge, MA 02139

**Keywords:** mRNA therapuetics, modified nucleotides, translation, RNA structure, SHAPE

## Abstract

Messenger RNAs (mRNAs) encode information in both their primary sequence and their higher order structure. The independent contributions of factors like codon usage and secondary structure to regulating protein expression are difficult to establish as they are often highly correlated in endogenous sequences. Here, we used 2 approaches, global inclusion of modified nucleotides and rational sequence design of exogenously delivered constructs, to understand the role of mRNA secondary structure independent from codon usage. Unexpectedly, highly expressed mRNAs contained a highly structured coding sequence (CDS). Modified nucleotides that stabilize mRNA secondary structure enabled high expression across a wide variety of primary sequences. Using a set of eGFP mRNAs with independently altered codon usage and CDS structure, we find that the structure of the CDS regulates protein expression through changes in functional mRNA half-life (i.e., mRNA being actively translated). This work highlights an underappreciated role of mRNA secondary structure in the regulation of mRNA stability.

## Significance

Despite widespread recognition that RNA is inherently structured, the interplay between local and global mRNA secondary structure (particularly in the coding region) and overall protein expression has not been thoroughly explored. Our work uses 2 approaches to disentangle the regulatory roles of mRNA primary sequence and secondary structure: global substitution with modified nucleotides and computational sequence design. By fitting detailed kinetic expression data to mathematical models, we show that secondary structure can increase mRNA half-life independent of codon usage. These findings have significant implications for both translational regulation of endogenous mRNAs and the emerging field of mRNA therapeutics.

Messenger RNAs (mRNAs) direct cytoplasmic protein expression. How much protein is produced per mRNA molecule is a function of how well the translational machinery initiates and elongates on the coding sequence (CDS) and the mRNA’s functional half-life. Both translational efficiency and functional half-life are driven by features encoded in the primary mRNA sequence. Synonymous codon choice directly impacts translation, with highly expressed genes tending to include more “optimal” codons (1, 2). Conversely, “nonoptimal” codons can increase ribosomal pausing and decrease mRNA half-life (3, 4). Other mRNA sequence features that reportedly correlate with protein output are dinucleotide frequency in the CDS (5) and the effect of codon order on locally accessible charged tRNA pools (6). Because these effects are interdependent on mRNA sequence, teasing apart their individual contributions to protein output is difficult and often controversial (7, 8).

In addition to dictating encoded protein identity, its primary sequence also determines an mRNA’s propensity to form secondary and tertiary structure (9). Transcriptome-wide RNA structure characterization is beginning to reveal global relationships between the structure content in different mRNA regions and protein expression (10, 11). Multiple studies have shown that secondary structure in the 5′ untranslated region (5′ UTR) generally reduces translation initiation efficiency and therefore overall protein output (10–13). But, the extent to which CDS and 3′ untranslated region (3′ UTR) secondary structure impacts protein output, however, is much less understood.

One way to alter RNA secondary structure is to change the primary sequence. In the CDS, however, primary sequence changes necessarily alter codon usage, confounding any effects that might be attributable to changes in mRNA structure alone. An alternate means to affect secondary structure without changing codons is to incorporate modified nucleotides (nt) that maintain the same Watson–Crick base-pairing relationships (e.g., pseudouridine [Ψ] for U) but have small effects on local secondary structure. Such modified nucleotides can either stabilize (14) or destabilize (15) base pairs and hence overall mRNA structure.

Here, we combined computational sequence design with global modified nucleotide substitution as tools to investigate the separate impacts of mRNA primary sequence and structural stability on protein output. We find that differences in the innate thermodynamic base pair stability of 2 modified uridine nucleotides, N^1^-methyl-pseudouridine and 5-methoxy-uridine, induce global changes in mRNA secondary structure. These structural changes in turn drive changes in protein expression. As expected, our data confirm that reduced secondary structure within a 5′ leader region (the 5′ UTR and first ∼10 codons of the CDS) correlates with high protein expression. Surprisingly, we also find that high protein expression correlates with increased secondary structure in the remainder of the mRNA (the rest of the CDS and the 3′ UTR). We validated this finding by designing an enhanced gene fluorescent protein (eGFP) mRNA panel wherein the effects of codon usage and secondary structure could be examined separately. Our data reveal a relationship wherein codon optimality and greater CDS secondary structure synergize to increase mRNA functional half-life.

## Results

### RNA Sequence and Nucleotide Modifications Combine to Determine Protein Expression.

**Fig. 1.**
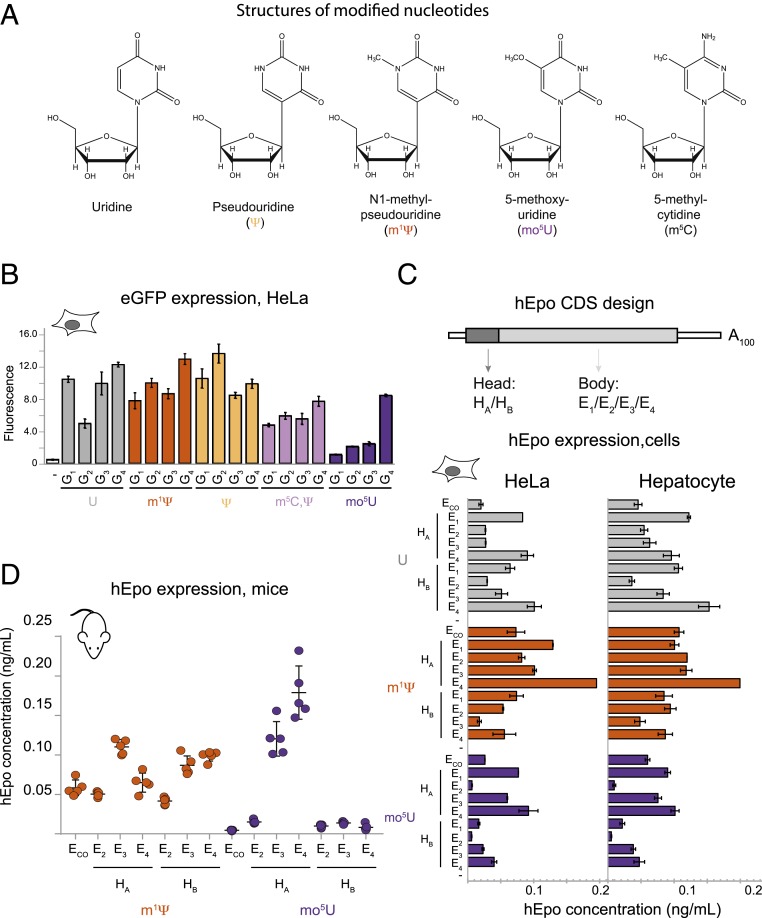
Inclusion of modified nucleotides in mRNA alters eGFP and hEPO expression. (*A*) Chemical structure of uridine and 4 modified nucleosides: (Ψ, m^1^Ψ, mo^5^U, and m^5^C. (*B*) Fluorescence intensity (normalized intensity units, *y* axis) of HeLa cells following transfection with lipofectamine alone (−) or 4 different eGFP sequence variants (G_1_–G_4_, *x* axis) containing uridine (gray), m^1^Ψ (orange), Ψ (yellow), m^5^C/Ψ (lavender), or mo^5^U (dark purple). (*C*, *Top*) Schematic of the hEpo mRNA sequence variants. Eight hEpo sequences combined 1 of 2 “head” regions (dark gray box, H_A_ or H_B_) encoding the first 30 amino acids (90 nucleotides) and 1 of 4 “body” regions (light gray box, E_1_–E_4_) encoding the remainder of the hEpo CDS. (*C*, *Bottom*) Levels of secreted hEpo protein measured by ELISA (ng/mL, *y* axis) following transfection of cells with 8 sequence variants (described in *B* above, *x* axis) plus 1 “codon optimized” variant (E_CO_) (44) containing uridine (gray bars), m^1^Ψ (orange), or mo^5^U (dark purple). (*D*) Serum concentrations of hEpo protein measured by ELISA (ng/mL, *y* axis) in BALB-c mice (5 per group) following IV injection of LNP-formulated mRNA of 6 sequence variants (described in *B* above, *x* axis) plus 1 codon optimized variant (E_CO_) (44) containing m^1^Ψ (orange) or mo^5^U (dark purple). Individual animals (dots) with mean and SE (black lines).

For this study, we created diverse synonymous CDS sets encoding eGFP (4 variants), human erythropoietin (hEpo, 9 variants) and firefly Luciferase (Luc, 39 variants) transcribed in vitro with ATP, CTP, GTP, and either UTP, pseudouridine triphosphate (ΨTP), N^1^-methyl-pseudouridine triphosphate (m^1^ΨTP), or 5-methyoxy-uridine triphosphate (mo^5^UTP) (Fig. 1*A*). For comparison with a previous study documenting the effects of modified nucleotides on RNA immunogenicity (16), we also made eGFP mRNA wherein both U and C were substituted with Ψ and 5-methyl-cytidine (m^5^C), respectively. We designed the sequence sets with bias toward optimal codons (for hEPO and eGFP mRNAs) or designed to sample a larger sequence space, including nonoptimal codons (for Luc mRNA). All mRNAs carried cap1, identical 5′ and 3′ UTRs, and a 100-nucleotide poly(A) tail.

**Fig. 2.**
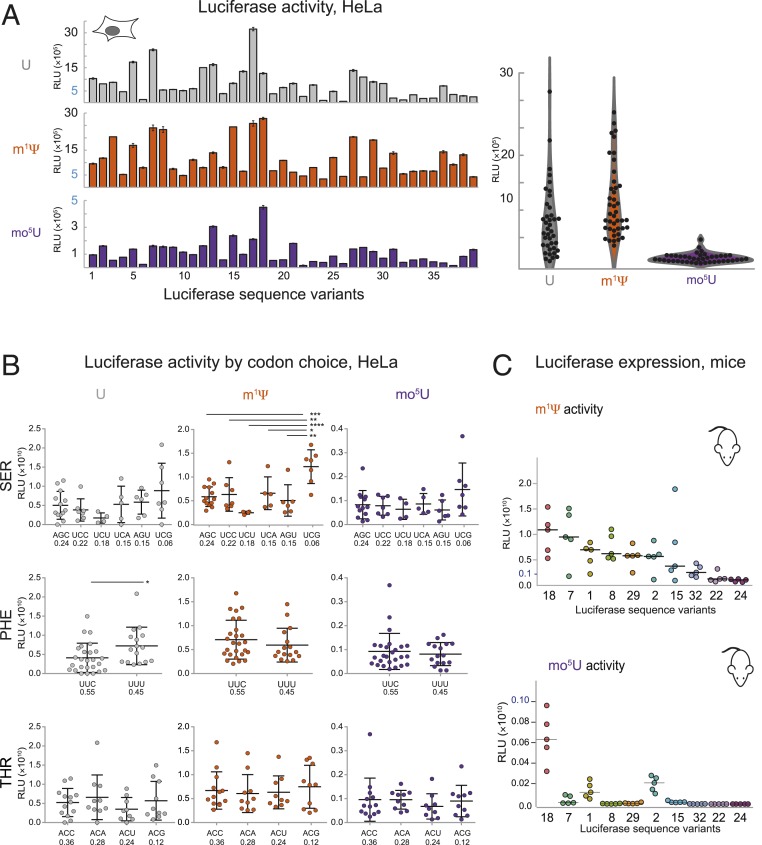
Inclusion of modified nucleotides in mRNA alters Luc expression. (*A*, *Left*) Expression in HeLa cells (relative light units [RLU], *y* axis) for 39 firefly Luciferase sequence variants (L_1_–L_39_, *x* axis) containing uridine (gray, *Top*), m^1^Ψ (orange, *Middle*), or mo^5^U (dark purple, *Bottom*). (*A*, *Right*) Distribution of expression levels (RLU, *y* axis) for variants (black dots) containing uridine (gray), m^1^Ψ (orange), and mo^5^U (dark purple) as a violin plot. (*B*) Expression in HeLa cells (RLU, *y* axis) of 39 firefly Luciferase variants grouped by the codon used (*x* axis) for all instances of serine (*Top*), phenylalanine (*Middle*), and threonine (*Bottom*) in mRNAs containing uridine (*Left*), m^1^Ψ (*Middle*), or mo^5^U (*Right*). Codons are shown in order of frequency of occurrence in the human transcriptome. Individual values (dots) are the same as in *A* with mean and SEs (black lines). Significant differences by 2-way ANOVA comparisons are indicated by lines above, and *P* values are noted by asterisks (*P ≤ 0.05, **P ≤ 0.01, ***P ≤ 0.001, and ****P ≤ 0.0001). (*C*) Total luminescence of in vivo firefly Luciferase expression (RLU, *y* axis) in CD-1 mice (5 per group) following IV injection of 0.15 mg/kg LNP-formulated mRNA for 10 sequence variants (*x* axis) containing m^1^Ψ (*Top*) or mo^5^U (*Bottom*). Individual animals (dots) with median.

First, we analyzed the impact of primary CDS sequence on protein expression of mRNAs containing no modified nucleotides (eGFP/hEPO, Fig. 1; Luc, Fig. 2). Cellular protein expression ranged between >2.5-fold for eGFP (Fig. 1*B*, gray) and >4-fold for hEpo (Fig. 1*C*, gray), despite all sequences containing only frequently used codons. Expression of 39 unmodified Luc variants containing codons with a greater optimality range varied >10-fold (Fig. 2*A*, gray). Highly expressed mRNAs tended to have increased GC content, consistent with previous reports (17), but not all high GC sequences were high expressers (*SI Appendix*, Figs. S1 *A* and *B* and S2*A*, gray). Unmodified Luc expression moderately correlated with both GC content and codon adaptation index (CAI) (Pearson correlations *r* = 0.63 and 0.64, respectively; see *SI Appendix*, Fig. S2*A*, gray). Each Luc variant globally used the same single codon for all instances of a given amino acid. This allowed us to look at the impact of individual codons on protein expression. Only 4 of 87 pairwise synonymous codon comparisons exhibited statistically significant differences (*P* < 0.05; see *SI Appendix*, Fig. S3, gray). For example, inclusion of Phe^UUU^ was associated with a slight increase in expression over Phe^UUC^ (Fig. 2*B*, gray). Surprisingly, even global inclusion of extremely nonoptimal codons such as Ser^UCG^, Leu^CUA^, Ala^GCG^, and Pro^CCG^ had no statistically significant impact on Luc expression in unmodified RNA (Fig. 2*B*, gray; see *SI Appendix*, Fig. S3*A*, gray). Thus, codon usage, as measured by metrics such as CAI, cannot adequately explain these data.

Next, we examined how protein expression was affected by global substitution with modified nucleotides in the same sequences. For eGFP mRNAs in HeLa cells, modified nucleotides changed the expression of both individual variants and the overall expression mean and range of the entire sequence set. Compared to unmodified mRNA, mean expression was similar for eGFP mRNAs containing Ψ and m^1^Ψ, but lower for mo^5^U and Ψ/m^5^C (3-fold and 1.5-fold lower, respectively, Fig. 1*B*). Of note, the identities of the best and worst expressing sequences were not consistent across the different modified nucleotides. For example, eGFP sequence G_2_ expressed highly with Ψ and m^1^Ψ, moderately with U and Ψ/m^5^C, but poorly with mo^5^U (Fig. 1*B*). Similar trends were observed for hEpo mRNA in HeLa cells, with m^1^Ψ yielding a 1.5-fold greater mean expression than U, which was in turn 2-fold higher than mo^5^U (Fig. 1*C*). As with eGFP and Luc, we observed hEpo variants (e.g., E_CO_ and H_A_E_2_) that expressed well with m^1^Ψ, but not U or mo^5^U-containing mRNA (Fig. 1*C*). Although we observed some variation in the expression levels of individual RNAs in hepatocytes versus HeLa cells, the general expression trends were remarkably similar (Fig. 1*C* and *SI Appendix*, Fig. S1*C*).

To extend this analysis, we next examined 39 synonymous Luc sequences containing U, m^1^Ψ, or mo^5^U mRNA in HeLa, AML12, and primary hepatocyte cells. Mean expression increased 1.5-fold for m^1^Ψ mRNA but decreased 5-fold for mo^5^U compared to unmodified mRNA in HeLa cells (Fig. 2*A*). This trend held in AML12 cells and primary hepatocytes cells as well as across delivery methods, including electroporation and transfection of lipid nanoparticles (LNPs) (although some individual differences were noted (Fig. 2*A* and *SI Appendix*, Figs. S2*B* and S3*B*). For several mRNA sequences, inclusion of modified nucleotides substantially impacted protein expression (Fig. 2*A* and *SI Appendix*, Fig. S2*C*). Several sequences (e.g., L_24_, and L_22_) universally produced low levels of protein across all modified nucleotides, but many variants (e.g., L_18_, L_7_, L_2_, L_8_, and L_29_) favored specific modified nucleotides over others. Taken together, these data indicate that sequence and nucleotide modifications make distinct contributions to the overall level of protein expression.

A simple explanation for the observed modified nucleotide-specific expression differences would be a direct effect on decoding by the ribosome. If so, expression should correlate with overall modified nucleotide content, or alternatively with the use of specific codons containing modified nucleotides. However, there is no clear relationship between % U content and expression (*SI Appendix*, Fig. S2*A*) and only a few m^1^Ψ- and mo^5^U-containing codons had any statistically significant impact on protein output (6 and 4, respectively, of 87 synonymous pairwise comparisons *P* < 0.05, Fig. 2*B* and see *SI Appendix*, Fig. S3*A*). A notable exception is an unexpected and unexplained 2-fold increase in protein production with inclusion of the nonoptimal codon Ser^UCG^ in m^1^Ψ mRNA (*P* < 0.05, Fig. 2*B* and see *SI Appendix*, Fig. S3*A*). Thus, mRNAs containing modified nucleotides (Ψ, m^1^Ψ, or mo^5^U) can support high levels of protein expression, but in a very context-specific manner.

To assess the degree to which the above conclusions from cell lines translated to animals, we examined protein expression in mice from formulated hEpo and Luc mRNA variants containing 2 nucleotide modifications shown to have reduced immunogenicity (m^1^Ψ and mo^5^U) (16). Unmodified mRNAs were not included because in vivo protein expression can be obscured by strong activation of innate immunity (18). For some hEpo mRNAs, such as m^1^Ψ H_B_E_3_, we noted expression differences between the cell lines and mice (Fig. 1 *C* and *D*). These differences were larger than the differences observed between cell lines, and more pronounced for m^1^Ψ hEPO mRNA than for mo^5^U hEPO mRNA (*SI Appendix*, Fig. S1*D*). However, general expression trends were maintained in vivo (Fig. 1*D*). All 6 sequence variants containing m^1^Ψ expressed well (Fig. 1*D*, orange), but only 2 containing mo^5^U mRNA expressed at detectable levels (Fig. 1*D*, purple). Further, the codon optimized variant E_CO_ expressed well with m^1^Ψ but not at all in mo^5^U. Even so, the best expression came from sequence variants containing mo^5^U (H_A_E_4_ and H_A_E_3_). The mo^5^U H_A_E_4_ variant produced >1.5-fold more protein than the best expressing m^1^Ψ variant (H_A_E_3_, Fig. 1*D*).

The 10 Luc variants tested in vivo were chosen to represent the widest possible range of protein expression observed in cell culture. As expected from the known biodistribution of mRNA-containing lipid nanoparticles (19), the liver was the main site of protein expression (*SI Appendix*, Fig. S2*E*). Luc mRNAs containing m^1^Ψ were highly expressed in vivo, particularly L_18_ and L_7_ (Fig. 2 *C*, *Top*). Variability in protein expression with mo^5^U was more exaggerated in vivo, as 7 of the 10 variants produced little to no protein (Fig. 2 *C*, *Bottom*). L_18_ was an exception, but still produced >10-fold less Luc than the same sequence with m^1^Ψ. Notably, L_7_ produced large amounts of protein with m^1^Ψ but barely detectable levels with mo^5^U (Fig. 2 *C*, *Top* versus Fig. 2 *C*, *Bottom*; note the *y* axis scales). These data suggest that expression differences observed in cell culture persist and can be more pronounced for exogenous RNAs delivered in vivo (*SI Appendix*, Fig. S2*D*).

### Protein Expression Differences Trends with mRNA Thermodynamic Stability.

**Fig. 3.**
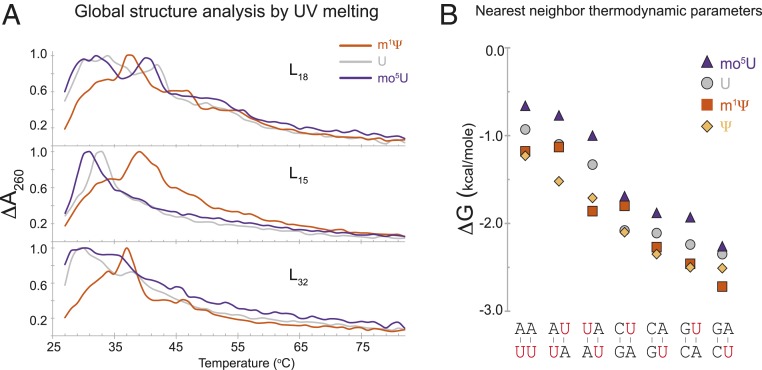
Modified nucleotides induce global structural changes in mRNA. (*A*) Optical melting profiles of firefly Luciferase sequence variants (L_18_ *Top*, L_15_ *Middle*, and L_32_ *Bottom*) containing uridine (gray), m^1^Ψ (orange), or mo^5^U (dark purple) showing the change in UV absorbance at 260 nm (*y* axis) as a function of temperature (*x* axis). (*B*) Nearest-neighbor thermodynamic parameters for Watson–Crick base pairs (*x* axis) containing uridine (gray circles, values from ref. 20), Ψ (yellow diamonds), m^1^Ψ (orange squares), or mo^5^U (dark purple triangles). The modified nucleotides in each nearest neighbor are highlighted in red.

Since codon usage alone could not fully explain sequence-dependent expression differences in mRNAs containing modified nucleotides, we examined how modified nucleotides might affect mRNA secondary structure. We determined UV absorbance melting curves for mRNAs across a range of expression levels containing different uridine analogs (U, m^1^Ψ, and mo^5^U) as an overall measure of secondary structure. Highly expressing mRNAs underwent substantial melting transitions, detected as sharp peaks in the melting curves, above 35 °C (e.g., variant L_18_ with all 3 uridine analogs and L_15_ with m^1^Ψ only; Fig. 3*A*). For some variants (e.g., L_15_), inclusion of m^1^Ψ but not mo^5^U induced a shift to higher melting temperatures, suggesting global stabilization of structural features within the mRNA (Fig. 3*A*). Notably, L_15_ expression was much higher with m^1^Ψ than mo^5^U (Fig. 2*A*). Similar trends were observed in most, but not all, sequences tested (*SI Appendix*, Fig. S4*A*). Although these initial results suggested a link between RNA structural stability and modification-dependent protein expression in vivo, higher resolution structural information was required.

The RNA base-pairing thermodynamics is commonly understood in nearest-neighbor energy terms (20). Whereas these parameters were previously reported for unmodified RNA and RNA containing Ψ, to our knowledge they have not yet been established for m^1^Ψ or mo^5^U. To establish these parameters for Ψ, m^1^Ψ, and mo^5^U, we performed optical melting experiments on 35 synthetic short RNA duplexes containing global substitutions of uridine with Ψ, m^1^Ψ, and mo^5^U (20). Nearest neighbors containing Ψ (Fig. 3*B*, diamonds) and m^1^Ψ (Fig. 3*B*, squares) form substantially more stable base pairs than uridine (by 0.25 and 0.18 kcal/mol on average, respectively; Fig. 3*B*, circles; *SI Appendix*, Table S1). In contrast, nearest neighbors containing mo^5^U (Fig. 3*B*, triangles) are destabilized by 0.28 kcal/mol relative to uridine (Fig. 3*B* and *SI Appendix*, Table S1). The average difference for mo^5^U versus Ψ is −0.5 kcal/mol per nearest neighbor, or −1.0 kcal/mol per base pair. The impact of each nucleotide modification on RNA is consistent across the nearest-neighbor base pairs when compared to U. This differs from a previous study (21) that found large context-dependent differences in energies of single A-Ψ pairs, depending on the flanking A-U and G-C pairs (*SI Appendix*, Fig. S4*B*), suggesting that introduction of single modified nucleotides can have complex, context-dependent impacts on folding energies. The global differences in pairing energies that we measured, summed over all base pairs, including a modified nucleotide in a full-length mRNA, readily explain the observed differences in the UV melting curves caused by inclusion of different modified nucleotides.

### Position-Dependent Structure Correlates with High Expression.

To investigate how modified nucleotides impact mRNA structure at single nucleotide resolution, we used selective 2’-hydroxyl acylation analyzed by primer-extension - mutational profiling (SHAPE-MaP) to probe RNA structure (22). We first verified that the methodology would produce high-quality data with m^1^Ψ and mo^5^U containing mRNAs (*SI Appendix*, Fig. S5*A*). In the absence of the SHAPE reagent (1-methyl-6-nitroisatoic anhydride [1M6]), there was no evidence of increased background error rates by next-generation sequencing (NGS) with either m^1^Ψ or mo^5^U (*SI Appendix*, Fig. S5*B*). The 1M6 treatment increased the mutation rates for RNAs containing either m^1^Ψ or mo^5^U to a similar extent as observed for uridine (*SI Appendix*, Fig. S5*C*). A comparison of SHAPE-induced mutation rates at U bases revealed a trend with m^1^Ψ < U < mo^5^U, which is consistent with the expected pairing frequency from the thermodynamic pairing energies (*SI Appendix*, Fig. S5*D*). Next, we measured RNA structure across the experimentally tested variants of hEpo containing U, m^1^Ψ, or mo^5^U (Dataset S1). Data for a representative sequence, hEpo H_A_E_3_, revealed local structure that differed dramatically by modified nucleotide (*SI Appendix*, Fig. S5 *A* and *D*). Consistent with the thermodynamic melting data in many RNAs, m^1^Ψ stabilized and mo^5^U destabilized structure (hEpo H_A_E_3_; see *SI Appendix*, Fig. S5 *D* and *E*). SHAPE-directed modeling of secondary structure suggested that modified nucleotides can induce widespread changes to the secondary structure ensemble for the same sequence (*SI Appendix*, Fig. S6 *A* and *B*). Thus, global incorporation of modified nucleotides induces widespread changes in mRNA structural content and conformation.

**Fig. 4.**
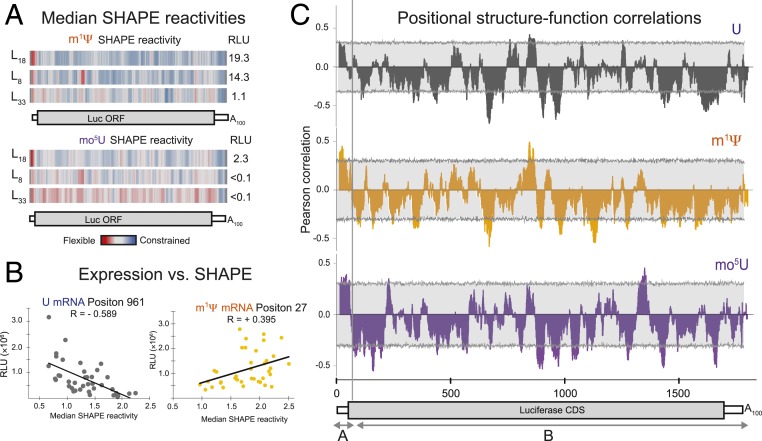
SHAPE data reveal a bipartite relationship between mRNA structure and protein expression. (*A*) Median SHAPE reactivity values (33-nt sliding window) for firefly Luc sequence variants L_18_ (*Top*), L_8_ (*Middle*), and L_32_ (*Bottom*), containing m^1^Ψ (orange, *Top*) or mo^5^U (purple, *Bottom*) shown as a heatmap: highly reactive (red), moderately reactive (gray), and lowly reactive (blue). Total luminescence values in mice from Fig. 2*C* are shown at *Right*. (*B*) Expression in HeLa cells (*y* axis, from Fig. 2*A*) for 39 firefly Luciferase variants (dots) containing uridine (dark gray, *Top*) or m^1^Ψ (orange, *Bottom*) versus median windowed SHAPE reactivity value (*x* axis) in 2 33-nt windows centered at the indicated positions. (*C*) Pearson correlations for SHAPE vs. protein expression in HeLa cells (*y* axis) across nucleotide positions (*x* axis) for 39 firefly Luciferase sequence variants containing U (dark gray, *Top*), m^1^Ψ (orange, *Middle*), or mo^5^U (dark purple, *Bottom*). The light gray boxes show the empirical 95% confidence interval at each position.

We next investigated the positional dependence of protein expression on local RNA structure. To do so, we obtained SHAPE data for synonymous variants of hEpo (8 variants each with m^1^Ψ or mo^5^U) and Luc (38 variants each with U, m^1^Ψ, or mo^5^U) whose protein output varied over >2 orders of magnitude (130 mRNAs total) (23). Regions displaying structural differences were identified using 31-nucleotide sliding window median reactivities, as previously described (24). Consistent with observations above, high protein output mRNA variants had lower median SHAPE reactivities (i.e., increased structure) across the CDS than low protein output variants. This was true for both proteins and all 3 chemistries (Fig. 4 *A* and *B* and Dataset S1). Particularly striking examples were E_CO_ and L_8_ mRNAs, where their high expression in m^1^Ψ compared to mo^5^U correlated with widespread m^1^Ψ-dependent decreases in median SHAPE reactivity throughout the CDS (Fig. 4*A* and *SI Appendix*, Fig. S5*E*). In contrast, the 5′ UTR of high-expressing variants exhibited high SHAPE, indicating a general lack of structure in this region (Fig. 4*A* and *SI Appendix*, Fig. S5*E*).

We next analyzed the directionality and strength of the correlation between positionwise SHAPE reactivity and protein expression across all Luc variants (Fig. 4*B*). This revealed a striking, position-dependent relationship between mRNA structure and expression that was largely consistent between mRNAs with m^1^Ψ and mo^5^U. The region encompassing the 47-nt 5′ UTR and the first ∼30 nucleotides of the CDS (Fig. 4*C*, region A) showed a positive and statistically significant correlation (*P* < 0.05) between SHAPE reactivity and protein expression for both m^1^Ψ and mo^5^U mRNAs. In contrast, the remainder of the CDS and the entire 3ʹ UTR (Fig. 4*C*, region B) exhibited a predominantly inverse correlation between SHAPE reactivity and protein expression for U, m^1^Ψ, and mo^5^U. For all modified nucleotides, the percent of nucleotides with negative correlations far outnumber those positions with positive correlations (U: 78.6%; m^1^Ψ: 78.7%; and mo^5^U: 72.9%) (Fig. 4*C*). In other words, increased secondary structure in these regions correlated with improved protein expression, consistent with the global structural properties measured by optical melting. Although statistically underpowered, a similar trend of higher protein expression from structured coding sequences was evident in the hEPO data (*SI Appendix*, Fig. S7). Notably, however, the strength of the structure–function correlation varied across the metasequence. Specific regions of the CDS exhibited statistically significant correlations between SHAPE reactivity and protein expression correlations of which the vast majority were negative rather than positive (U: 1 positive, 15 negative; m^1^Ψ: 1 positive, 18 negative; and mo^5^U: 2 positive, 17 negative) (Fig. 4*C*).

**Fig. 5.**
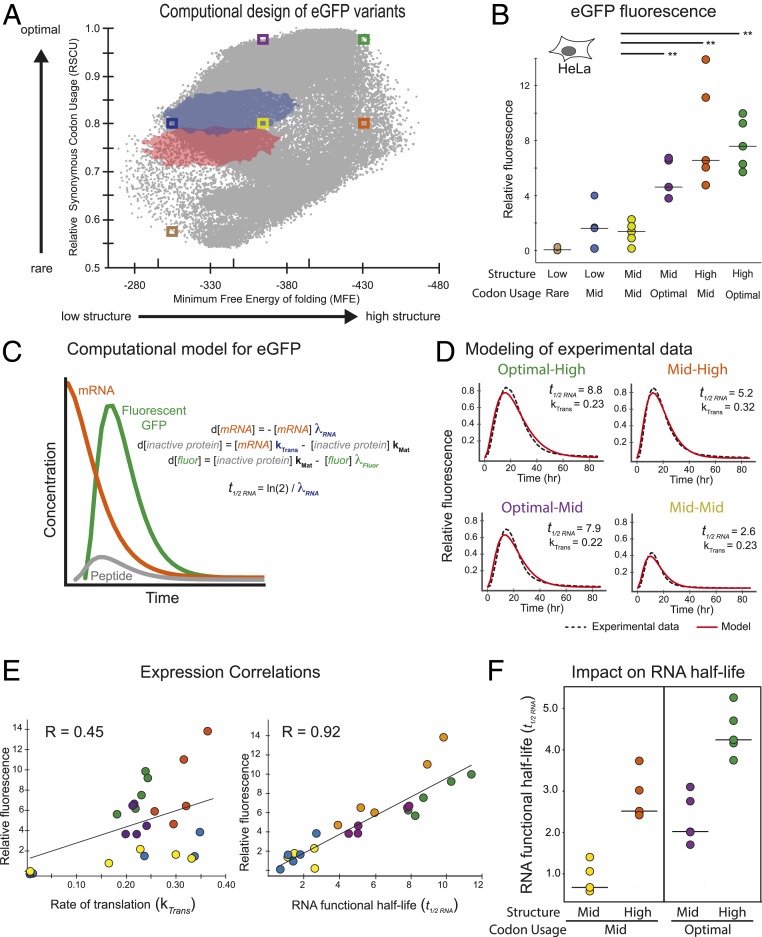
Half-life of computationally designed eGFP-degron mRNAs is determined by codon usage and mRNA structure. (*A*) Codon optimality (relative synonymous codon usage, *y* axis) versus secondary structure (energy of the predicted MFE structure, *x* axis) for sets of 150,000 generated eGFP sequence variants generated using codons chosen randomly (red), weighted in proportion to the human genome(blue), and using our algorithm (gray). Colored boxes show regions from which sequences were selected for further testing. (*B*) Total integrated eGFP fluorescence measured every 2 h for 86 h in HeLa cells (relative fluorescence unit [RFU], *y* axis) for 6 sets of 5 mRNAs containing m^1^Ψ (dots, with median as black line) with differing degrees of codon optimality and/or secondary structure (*x* axis, as in *A*). Significant differences by 2-way ANOVA comparisons are indicated by lines above, and *P* values are noted by asterisks (**P ≤ 0.01). (*C*) Model of eGFP expression kinetics. Simulated curves based on equations for changes in levels of mRNA (mRNA, orange), immature nonfluorescent protein (inactive protein, gray), and mature fluorescent protein (fluor, green) over time using exponential decay rates for mRNA (λ_RNA_) and eGFP protein (λ_Fluor_), and rates of translation (k_Trans_) and protein maturation (k_MAT_). mRNA half-lives (*t*_*1/2*__*RNA*_) were calculated from the observed mRNA decay rates. (*D*) eGFP-degron fluorescence in HeLa cells (RFU, *y* axis) versus time (*x* axis) as measured experimentally (solid colored lines as in *A*) and fitted according to the model in *C* (dashed black lines) for representative sequence variants with differing degrees of codon optimality and/or secondary structure (as in *A*). Translation rate constants (k_Trans_) and mRNA half-lives (*t*_*1/2*__*RNA*_) as derived from the model described in *C* are shown. (*E*) Total eGFP-degron fluorescence in HeLa cells (RFU, *y* axis) versus the modeled rate constants for translation (k_Trans_, *Left*) or mRNA functional half-life (λ_RNA_, *Right*) for 20 sequence variants containing m^1^Ψ as in D. Linear regression (black line) and Pearson correlation are shown. (*F*) Modeled functional mRNA half-lives (λ_RNA_, *y* axis) for 4 sets of 5 eGFP-degron sequence variants with differing degrees of codon optimality and/or secondary structure (*x* axis, as in *A* and *B*).

The observed structure–function relationships were evaluated further using targeted mutations. We examined the role of flexibility in region A (47-nt 5′ UTR and the first 30 nucleotides of the CDS) by creating chimeras combining variants with different structural signatures. Luc variants L_7_ and L_27_ (Fig. 2*A*) both exhibited lower than average protein expression in m^1^Ψ. Both also exhibited low SHAPE reactivity (high structure) throughout both regions A and B (Dataset S1). However, when we replaced region A with the relatively unstructured corresponding region A from the high expresser L_18_ (Fig. 4*A*) to produce fusion mRNAs FL_18/7_ and FL_18/27_, both region A SHAPE reactivity and Luc expression increased (*SI Appendix*, Fig. S8*A* and Fig. 5*B*). The FL_18/7_ and FL_18/27_ chimeras only differed by 2 and 4 individual bases from their respective parents (note that the 47-nucleotide 5′ UTR is common to all sequences). Consistent with the structure–function correlations within region B (the rest of the CDS and the 3′ UTR), a Luciferase variant (L_HS_) predicted to have more stable secondary structure in CDS (L_HS_ for high structure) yielded 1.5-fold greater protein expression than L_18_ in mo^5^U (*SI Appendix*, Fig. S8*C*). The expression of L_HS_ in m^1^Ψ was slightly lower than L_18_ (*SI Appendix*, Fig. S8*C*), likely due to more stable secondary structure near the start codon (*SI Appendix*, Fig. S8*D*). While this is consistent with our observation that stable CDS structure correlates with increased protein expression, a more rigorous approach was needed to disentangle the role of structure from codon optimality.

### Codon Usage and mRNA Structure Synergize to Determine Ribosome Loading and mRNA Half-Life.

The redundancy of the genetic code means that it is impossible to completely enumerate the relationships between codon usage, mRNA secondary structure, and protein expression. Instead we computationally generated sets of 150,000 synonymous CDSs encoding eGFP-degron with 3 different algorithms. For each sequence, we calculated relative synonymous codon usage (RSCU, ref. 25) and the predicted minimum free energy (MFE) structure (26). Randomly choosing synonymous codons with equal probability generates sequences that cluster around 0.75 ± 0.05 RSCU and −325 ± 40 kcal/mol MFE (Fig. 5*A*, red). Using probabilities weighted by frequency in the human transcriptome (27) generates a similar-shaped distribution, but shifted to both significantly higher RSCU (0.825 ± 0.05, *P* < 0.05) and greater structure (−340 ± 40 kcal/mol, *P* < 0.05) (Fig. 5*A*, blue). Next, we developed an algorithm that varied the individual codon choice probabilities dynamically so that RCSU and MFE were both driven to their accessible extremes (Fig. 5*A*, gray). The space covered is far greater than for random or frequency-weighted sequences, but has well-defined limits. Notably, because optimal codons tend to be GC rich the structure of the genetic code inherently disallows sequences with both highly optimal codons and low structure (Fig. 5 *A*, *Top* *Left* corner) or rare codons and high structure (Fig. 5 *A*, *Bottom Right* corner).

To investigate how the limits of codon optimality and allowable structure affect protein expression, we selected 6 regions collectively spanning the range of accessible space (Fig. 5*A*, boxes). From each of these 6 selected regions, we synthesized 5 synonymous sequences (30 in total) and followed the production and decay of GFP fluorescence over a 20-h timecourse in HeLa cells. This enabled us to directly compare the effects changing each factor independently, for example changes in MFE at constant RSCU (Fig. 5*A*, yellow vs. orange or purple vs. green) or the converse (Fig. 5*A*, yellow vs. purple or orange vs. green). The calculated folding energies of a subset of the eGFP mRNAs were validated by obtaining both SHAPE data and data-directed folding models (*SI Appendix*, Fig. S9 *A* and *B*). As expected, eGFP-degron mRNAs containing rare codons and very little secondary structure produced minimal protein (Fig. 5*B*, brown). Low protein expression was also observed for mRNAs with middling scores in both relative synonymous codon usage and structure (Fig. 5*B*, yellow). Notably, increasing either the codon optimality or secondary structure while holding the other feature constant both increased median protein expression (Fig. 5*B*, orange and purple, respectively, *P* value < 0.01). The set of mRNAs with the highest codon optimality and most structure, however, showed no additional increase in median protein expression (Fig. 5*B*, green, *P* value = 0.55). Local percentages of both U and A gave similar negative correlation across the entire CDS (*SI Appendix*, Fig. S10). Similar effects were observed in AML12 cells (*SI Appendix*, Fig. S11*A*). Combined, these data indicate that codon usage and secondary structure are both important, but distinct regulators of overall protein expression.

Next, we analyzed the kinetics of protein production. Real-time, continuous expression data were fit by a model including rate constants for mRNA translation, mRNA functional half-life, maturation of eGFP protein into its fluorescent form (28), and eGFP protein degradation (Fig. 5*C*). Functional half-life reflects the productive life of the mRNA in generating protein and is not necessarily the same as physical half-life ending with degradation—it could also reflect intracellular trafficking or sequestration away from the ribosomal machinery. Since all mRNA sequences expressed the same eGFP protein sequence and we measured fluorescent (i.e., mature functional) protein, we could assume constant rates of protein maturation (k_Mat_) and protein degradation (λ_Fluor_, Fig. 5*C*). Fitting this model to the experimental data allowed us to calculate the rate of translation (k_Trans_) and functional half-life (t_1/2__RNA_) individually for each mRNA variant (Fig. 5*D* and *SI Appendix*, Table S2). Surprisingly, whereas overall protein expression correlated poorly with mRNA translation rate (*r* = 0.45), it correlated remarkably well with functional mRNA half-life (*r* = 0.90, Fig. 5*E*). Although the model was necessarily simplistic, these results were consistent across multiple computational models including models containing a delivery rate (*SI Appendix*, Fig. S11 *B* and *C*). Highly structured mRNAs had a >2-fold increase in functional mRNA half-life relative to those with middling degrees of secondary structure, regardless of whether their codon usage was middling or optimal (Fig. 5*F*). Thus, secondary structure increases protein output by extending mRNA functional half-life in a previously unrecognized regulatory mechanism independent of codon optimality.

## Discussion

The amount of protein produced from any given mRNA (i.e., the translational output) is influenced by multiple factors specified by the primary nucleotide sequence. These factors include GC content, codon usage, codon pairs, and secondary structure. Disentangling the individual roles played by each of these factors in translational output of endogenous mRNAs, however, has proven difficult because of their high covariance. To separate these confounding relationships, we directly manipulated the secondary structure of exogenously delivered mRNAs using 2 distinct approaches. First, we globally replaced uridine with modified analogs having markedly different base-pairing thermodynamics—this led to global secondary structure changes without altering the mRNA sequence. Second, we used computational design to identify sets of mRNAs whose coding sequences explored the limits of codon usage and secondary structure.

Global incorporation of different modified nucleotides often (but not always) markedly changed mRNA expression. This effect was seen across numerous synonymous coding variants of multiple proteins, in several different cell lines, and in vivo (Figs. 1 and 2). m^1^Ψ generally gave higher expression than U or mo^5^U for the same sequence. Biophysical studies revealed that m^1^Ψ and mo^5^U have dramatically different and opposite effects compared to U (stabilizing and destabilizing, respectively) on overall mRNA folding, nearest-neighbor base-pairing thermodynamics, and secondary structure pattern as mapped by SHAPE (Figs. 3 and 4). We also found that secondary structure correlates with protein expression in a position-specific manner (Fig. 4). Consistent with previous reports (10–13), highly expressed mRNAs had low structure in the entire 5′ UTR and the first ∼30 CDS nucleotides. Notably, even though the constant, 47-nucleotide 5′ UTR was chosen to support high expression across many coding sequences, we still observed a clear structure–expression relationship in this region. Unexpectedly, however, we found that a highly structured CDS region downstream of the first 30 nucleotides also correlates with increased protein expression. By rationally designing sequences to contain more CDS structure, we could rescue low-expressing mo^5^U-containing mRNA variants (*SI Appendix*, Fig. S8*A*). Protein expression from sequences selected to vary the degree of secondary structure independent of codon usage, and vice versa, revealed that secondary structure and codon usage each have distinct and roughly equivalent impacts on protein output (Fig. 5). For this set of mRNAs, total protein output was driven primarily by changes in functional mRNA half-life.

There are several possible mechanisms for the observed relationship between CDS secondary structure and functional mRNA half-life. It is possible that higher mRNA structure improves interaction with RNA binding proteins (RBPs) that positively impact translation, such as double-stranded RNA binding protein Staufen (29) or reduces accessibility to single strand-specific endonucleases, although endonucleolytic cleavage is thought to be limited to specific cases (30). Another possibility is that ribosome queuing near the start codon may facilitate translation initiation (31). This could explain the structural signature in the beginning of the CDS, but it is worth noting that our data suggest a beneficial effect for structure throughout the CDS (Fig. 4*C*). Another possibility is that structure leads to ribosome pausing at specific locations in the peptide, crucial for proper protein folding, and therefore activity, of certain multidomain proteins (32, 33).

Yet another possibility is that mRNA structure slows ribosome movement, leading to enhanced functional protein output and extended mRNA half-life. Biophysical experiments have shown that secondary structure can slow ribosome processivity (34). While counterintuitive, the increase in protein output is consistent with previous work in cell-free lysates, showing that mRNAs containing m^1^Ψ slow ribosome elongation, increase total protein output, and improve initiation through decreased phosphorylation of the initiation factor, eIF2α (35). Our data help explain the first 2 observations, in that m^1^Ψ stabilizes mRNA secondary structure leading to greater protein output. If eIF2α dephosphorylation were the primary driver of the change in translation, we would have expected to see a strong correlation between translation efficiency and total protein output. However, we found very little correlation between translation efficiency, which should closely correlate to initiation rate and total protein output (Fig. 5*E*). Our use of a standardized 5′ UTR lacking strong translational modulators found in many endogenous genes, such as upstream ORFs and specific regulatory structures, likely rule out effects of those elements. Interestingly, a recent study in mammalian neurons links codon-specific ribosome stalling directly to eIF2α phosphorylation through the kinase GCN2 (36), suggesting that translation initiation and elongation may be highly interconnected (37).

Further, ribosomal pauses induced by rare codons have recently been directly linked to ribosomal frame shifting (38) and mRNA degradation (4, 39). An mRNA degradation-based mechanism is consistent with our data showing a tight correlation between mRNA half-life and total protein output (Fig. 5*E*). Notably, ribosome collisions were demonstrated to activate the mRNA degradation through the no-go decay (NGD) pathway and decrease mRNA half-life (40, 41). One suggested mechanism for this regulation is the action of the ubiquitin ligase, ZNF598, on the small subunit proteins of collided diribosomes (42). An aspect of this regulation that remains poorly understood is the relationship between ribosomal pausing/collision and secondary structure. Although secondary structure likely slows ribosome progression, the ribosome is an inherently powerful helicase that necessarily unwinds mRNA structure during elongation (43). We propose that local secondary structure elements unwound by each advancing ribosome should quickly reform after that ribosome has moved on and may thus act as buffers to prevent collisions between adjacent ribosomes on the same message. If ribosome collisions play a central role in translational quality control, local secondary structure may be a key regulator of functional mRNA half-life by enforcing spacing between ribosomes and thereby decreasing collisions.

## Methods

### Animal Research.

All animal studies were approved by and performed in accordance with the Institutional Animal Care and Use Committee of Moderna, Inc.

### Sequence Design.

eGFP variants were stochastically generated using only frequently used codons. For hEpo regions and Luc, variants were deterministically encoded with one codon per amino acid.

### mRNA Preparation.

mRNAs for hEpo, eGFP, and Luc were synthesized in vitro using all unmodified nucleotides or global substitutions of uridine (U) for the modified uridine analogs pseudouridine (Ψ), N^1^-methyl-pseudouridine (m^1^Ψ), 5-methyoxy-urdine (mo^5^U), or a combination of Ψ and 5-methyl-cytidine (m^5^C).

### Determination of Nearest-Neighbor Thermodynamic Parameters.

UV-melting experiments were performed on 39 synthetic RNA duplexes with Ψ, m^1^Ψ, and mo^5^U instead of uridine, and the nearest-neighbor free energy contributions for each modified nucleotide to be determined using established methods (20).

### Computational Modeling of eGFP Expression Data.

Timecourse data were collected from HeLa and AML12 cells transfected with the designed eGFP-degron mRNAs. These data were used to fit the computational model of active protein production and degradation in which rate terms for protein maturation and degradation were held constant and the translation efficiency and rate of RNA degradation were allowed to vary to find the best fit to the experimental data.

## Supplementary Material

Supplementary File

Supplementary File

Supplementary File

## References

[1] Gustafsson C., Govindarajan S., Minshull J., Codon bias and heterologous protein expression. Trends Biotechnol. 22, 346–353 (2004). 15245907 10.1016/j.tibtech.2004.04.006

[2] Horstick E. J., Increased functional protein expression using nucleotide sequence features enriched in highly expressed genes in zebrafish. Nucleic Acids Res. 43, e48 (2015). 25628360 10.1093/nar/gkv035 PMC4402511

[3] Weinberg D. E., Improved ribosome-footprint and mRNA measurements provide insights into dynamics and regulation of yeast translation. Cell Rep. 14, 1787–1799 (2016). 26876183 10.1016/j.celrep.2016.01.043 PMC4767672

[4] Presnyak V., Codon optimality is a major determinant of mRNA stability. Cell 160, 1111–1124 (2015). 25768907 10.1016/j.cell.2015.02.029 PMC4359748

[5] Tulloch F., Atkinson N. J., Evans D. J., Ryan M. D., Simmonds P., RNA virus attenuation by codon pair deoptimisation is an artefact of increases in CpG/UpA dinucleotide frequencies. eLife 3, e04531 (2014). 25490153 10.7554/eLife.04531 PMC4383024

[6] Tuller T., An evolutionarily conserved mechanism for controlling the efficiency of protein translation. Cell 141, 344–354 (2010). 20403328 10.1016/j.cell.2010.03.031

[7] Simmonds P., Tulloch F., Evans D. J., Ryan M. D., Attenuation of dengue (and other RNA viruses) with codon pair recoding can be explained by increased CpG/UpA dinucleotide frequencies. Proc. Natl. Acad. Sci. U.S.A. 112, E3633–E3634 (2015). 26071447 10.1073/pnas.1507339112 PMC4507241

[8] Futcher B., Reply to Simmonds et al.: Codon pair and dinucleotide bias have not been functionally distinguished. Proc. Natl. Acad. Sci. U.S.A. 112, E3635–E3636 (2015). 26071446 10.1073/pnas.1507710112 PMC4507201

[9] Mortimer S. A., Kidwell M. A., Doudna J. A., Insights into RNA structure and function from genome-wide studies. Nat. Rev. Genet. 15, 469–479 (2014). 24821474 10.1038/nrg3681

[10] Ding Y., In vivo genome-wide profiling of RNA secondary structure reveals novel regulatory features. Nature 505, 696–700 (2014). 24270811 10.1038/nature12756

[11] Wan Y., Landscape and variation of RNA secondary structure across the human transcriptome. Nature 505, 706–709 (2014). 24476892 10.1038/nature12946 PMC3973747

[12] Shah P., Ding Y., Niemczyk M., Kudla G., Plotkin J. B., Rate-limiting steps in yeast protein translation. Cell 153, 1589–1601 (2013). 23791185 10.1016/j.cell.2013.05.049 PMC3694300

[13] Tuller T., Zur H., Multiple roles of the coding sequence 5′ end in gene expression regulation. Nucleic Acids Res. 43, 13–28 (2015). 25505165 10.1093/nar/gku1313 PMC4288200

[14] Newby M. I., Greenbaum N. L., A conserved pseudouridine modification in eukaryotic U2 snRNA induces a change in branch-site architecture. RNA 7, 833–845 (2001). 11424937 10.1017/s1355838201002308 PMC1370140

[15] Kierzek E., Kierzek R., The thermodynamic stability of RNA duplexes and hairpins containing N6-alkyladenosines and 2-methylthio-N6-alkyladenosines. Nucleic Acids Res. 31, 4472–4480 (2003). 12888507 10.1093/nar/gkg633 PMC169893

[16] Karikó K., Incorporation of pseudouridine into mRNA yields superior nonimmunogenic vector with increased translational capacity and biological stability. Mol. Ther. 16, 1833–1840 (2008). 18797453 10.1038/mt.2008.200 PMC2775451

[17] Plotkin J. B., Kudla G., Synonymous but not the same: The causes and consequences of codon bias. Nat. Rev. Genet. 12, 32–42 (2011). 21102527 10.1038/nrg2899 PMC3074964

[18] Kormann M. S., Expression of therapeutic proteins after delivery of chemically modified mRNA in mice. Nat. Biotechnol. 29, 154–157 (2011). 21217696 10.1038/nbt.1733

[19] Sabnis S., A novel amino lipid series for mRNA delivery: Improved endosomal escape and sustained pharmacology and safety in non-human primates. Mol. Ther. 26, 1509–1519 (2018). 29653760 10.1016/j.ymthe.2018.03.010 PMC5986714

[20] Xia T., Thermodynamic parameters for an expanded nearest-neighbor model for formation of RNA duplexes with Watson-Crick base pairs. Biochemistry 37, 14719–14735 (1998). 9778347 10.1021/bi9809425

[21] Hudson G. A., Bloomingdale R. J., Znosko B. M., Thermodynamic contribution and nearest-neighbor parameters of pseudouridine-adenosine base pairs in oligoribonucleotides. RNA 19, 1474–1482 (2013). 24062573 10.1261/rna.039610.113 PMC3851715

[22] Siegfried N. A., Busan S., Rice G. M., Nelson J. A., Weeks K. M., RNA motif discovery by SHAPE and mutational profiling (SHAPE-MaP). Nat. Methods 11, 959–965 (2014). 25028896 10.1038/nmeth.3029 PMC4259394

[23] Mauger D. M., Cabral B. J., Presnyak V., Moore M. J., mRNA structure regulates protein expression through changes in functional half-life. Gene Expression Omnibus (GEO) database. https://www.ncbi.nlm.nih.gov/geo/query/acc.cgi?acc=GSE139176. Deposited 21 October 2019. 10.1073/pnas.1908052116 PMC6883848 31712433

[24] Watts J. M., Architecture and secondary structure of an entire HIV-1 RNA genome. Nature 460, 711–716 (2009). 19661910 10.1038/nature08237 PMC2724670

[25] Scherer S., Guide to the Human Genome (Cold Spring Harbor Laboratory Press, Cold Spring Harbor, NY, 2010) p. xiv, 1008 p.

[26] Lu Z. J., Turner D. H., Mathews D. H., A set of nearest neighbor parameters for predicting the enthalpy change of RNA secondary structure formation. Nucleic Acids Res. 34, 4912–4924 (2006). 16982646 10.1093/nar/gkl472 PMC1635246

[27] Nakamura Y., Gojobori T., Ikemura T., Codon usage tabulated from international DNA sequence databases: Status for the year 2000. Nucleic Acids Res. 28, 292 (2000). 10592250 10.1093/nar/28.1.292 PMC102460

[28] Crameri A., Whitehorn E. A., Tate E., Stemmer W. P., Improved green fluorescent protein by molecular evolution using DNA shuffling. Nat. Biotechnol. 14, 315–319 (1996). 9630892 10.1038/nbt0396-315

[29] Jungfleisch J., A novel translational control mechanism involving RNA structures within coding sequences. Genome Res. 27, 95–106 (2017). 27821408 10.1101/gr.209015.116 PMC5204348

[30] Schoenberg D. R., Mechanisms of endonuclease-mediated mRNA decay. Wiley Interdiscip. Rev. RNA 2, 582–600 (2011). 21957046 10.1002/wrna.78 PMC3347869

[31] Kearse M. G., Ribosome queuing enables non-AUG translation to be resistant to multiple protein synthesis inhibitors. Genes Dev. 33, 871–885 (2019). 31171704 10.1101/gad.324715.119 PMC6601509

[32] Kimchi-Sarfaty C., A “silent” polymorphism in the MDR1 gene changes substrate specificity. Science 315, 525–528 (2007). 17185560 10.1126/science.1135308

[33] Rauscher R., Ignatova Z., Timing during translation matters: Synonymous mutations in human pathologies influence protein folding and function. Biochem. Soc. Trans. 46, 937–944 (2018). 30065107 10.1042/BST20170422

[34] Wen J. D., Following translation by single ribosomes one codon at a time. Nature 452, 598–603 (2008). 18327250 10.1038/nature06716 PMC2556548

[35] Svitkin Y. V., N1-methyl-pseudouridine in mRNA enhances translation through eIF2α-dependent and independent mechanisms by increasing ribosome density. Nucleic Acids Res. 45, 6023–6036 (2017). 28334758 10.1093/nar/gkx135 PMC5449617

[36] Ishimura R., Nagy G., Dotu I., Chuang J. H., Ackerman S. L., Activation of GCN2 kinase by ribosome stalling links translation elongation with translation initiation. eLife 5, e14295 (2016). 27085088 10.7554/eLife.14295 PMC4917338

[37] Chu D., Translation elongation can control translation initiation on eukaryotic mRNAs. EMBO J. 33, 21–34 (2014). 24357599 10.1002/embj.201385651 PMC3990680

[38] Simms C. L., Yan L. L., Qiu J. K., Zaher H. S., Ribosome collisions result in +1 frameshifting in the absence of no-go decay. Cell Rep. 28, 1679–1689.e4 (2019). 31412239 10.1016/j.celrep.2019.07.046 PMC6701860

[39] Radhakrishnan A., The DEAD-box protein Dhh1p couples mRNA decay and translation by monitoring codon optimality. Cell 167, 122–132.e9 (2016). 27641505 10.1016/j.cell.2016.08.053 PMC5635654

[40] Simms C. L., Yan L. L., Zaher H. S., Ribosome collision is critical for quality control during no-go decay. Mol. Cell 68, 361–373.e5 (2017). 28943311 10.1016/j.molcel.2017.08.019 PMC5659757

[41] D’Orazio K. N., The endonuclease Cue2 cleaves mRNAs at stalled ribosomes during no go decay. eLife 8, e49117 (2019). 31219035 10.7554/eLife.49117 PMC6598757

[42] Juszkiewicz S., ZNF598 is a quality control sensor of collided ribosomes. Mol. Cell 72, 469–481.e7 (2018). 30293783 10.1016/j.molcel.2018.08.037 PMC6224477

[43] Mustoe A. M., Pervasive regulatory functions of mRNA structure revealed by high-resolution SHAPE probing. Cell 173, 181–195.e18 (2018). 29551268 10.1016/j.cell.2018.02.034 PMC5866243

[44] Welch M., Design parameters to control synthetic gene expression in Escherichia coli. PLoS One 4, e7002 (2009). 19759823 10.1371/journal.pone.0007002 PMC2736378

