# Supplementary material for: mRNA structure regulates protein expression through changes in functional half-life
Source: Proc Natl Acad Sci U S A. 2019 Nov 11;116(48):24075–83. doi: 10.1073/pnas.1908052116 (PMC6883848; doi:10.1073/pnas.1908052116)
Supplement: Supplementary File [file pnas.1908052116.sapp.pdf]

PNAS
www.pnas.org

# Supplementary Information for

mRNA structure regulates protein expression through changes in functional half-life.

David M. Mauger[1], B. Joseph Cabral[1], Vladimir Presnyak[1], Stephen V. Su[1], David W. Reid[1], Brooke Goodman[1], Kristian Link[1], Nikhil Khatwani[1], John Reynders[1+], Melissa J. Moore[1*], and Iain J. McFadyen[1]
[1] Moderna Therapeutics, 200 Technology Square, Cambridge MA, 02139 USA

**Corresponding Authors:** Melissa J. Moore

Email: Melissa.Moore@Modernatx.com

**This PDF file includes:**

- Supplementary methods
- Supporting Figure Legends
- Supporting Table Legends
- Supporting Figures S1 to S11
- Supporting Table S1
- Supporting Table S2
- Supplementary References

**Other supplementary materials for this manuscript include the following:**

- Dataset 1 and Dataset 2



www.pnas.org/cgi/doi/10.1073/pnas.1908052116

**Supplementary Methods:**

**Experimental Model Details.**

Cell culture models:

HeLa (ATCC CCL-2), Vero (ATCC CCL-81), BJ (ATCC CRL-2522), HepG2 (ATCC HB-8065), and AML12 (ATCC CRL-2254) cells were maintained in Dulbecco's Modified Eagle's Medium (DMEM) supplemented with GlutaMAX, HEPES, high glucose (Life Technologies, cat. no. 10564-011), 10% fetal bovine serum (FBS) (Life Technologies, cat. no. 10082-147) and sodium pyruvate (Life Technologies, cat. no. 11360-070) at 37°C in a humidified incubator at 5% $CO_2$ atmosphere. Cells were passaged every 3-4 days with 0.25% trypsin-EDTA solution (Life Technologies, cat. no. 25200-056) and washed with sterile PBS (Life Technologies, cat. no. 10010-049) under aseptic conditions, for no more than 20 passages.

For all primary mouse hepatocytes *in vitro* assays, cryopreserved primary mouse hepatocytes (ThermoFisher cat. no. HMCPIS) were thawed and immediately plated for use in CHRM (ThermoFisher cat. no. CM7000), Williams Medium E supplemented with Hepatocyte Plating Supplement Pack (Serum-Containing). Plates were incubated at 37 °C in a humidified incubator at 5% $CO_2$ atmosphere for 5 hours before changing to serum free media (William's E Maintenance Media – Without Serum). Plates were incubated at 37 °C in a humidified incubator at 5% $CO_2$ atmosphere for all periods between active use.

Mice models:

*In vivo* protein expression experiments for hEpo and Luc mRNAs were performed using CD-1 and BALB/C mouse models.

**Method Details.**

<u>Sequence Design</u>

eGFP variants ($G_1$- $G_4$) were stochastically generated using only frequently used codons. For hEpo, we obtained one mammalian codon-optimized sequence variant ($E_{CO}$) (58) and eight variants generated by combining two unique sequences encoding the first 30 amino-acids ($H_A$, $H_B$) with four different variants of the remainder of the CDS ($E_1$, $E_2$, $E_3$, $E_4$). A larger set of Luc variants deterministically encoded each instance of a given amino acid throughout the coding sequence with the same single codon, with the set of 20 codons used differing between variants.

In all cases, the coding sequence was flanked by identical 5′ and 3′ untranslated regions (UTRs) capable of supporting high levels of protein expression (Figure 1B). Thus, total protein expression from these exogenous RNAs is determined by the combined impact of the primary coding sequence and the nucleotides used. For simplicity and ease of analysis, we designed firefly luciferase mRNA sequences based on simple one-to-one codon sets (i.e. each amino acid is encoded by the same codon at every instance of the amino acid (Table S3) that disfavored the use of rare codons). Regions of increased rare codon frequency have been shown to decrease protein expression and mRNA stability (1, 2). The hEpo protein contains a 9-amino acid (27 nucleotide) signal peptide sequence that is removed from the mature protein after targeting the protein to the endoplasmic reticulum (ER) for secretion. To evaluate whether codon choice had different effects in

the signal peptide region, we tested sequence designs for hEpo in which a leader region of 30 amino acids was encoded using two distinct codon sets: L1 (an AU-rich codon set) and L2 (a GC-rich codon set) (Figure 1C). eGFP mRNAs were computationally designed by an algorithm written to stochastically sample the sequence space of a given peptide sequence.

## mRNA Preparation:

mRNA was produced by in vitro transcription (IVT) using T7 RNA polymerase using a linearized DNA template encoding the RNA polymerase promotor followed by 5' UTR, ORF, 3'UTR, and poly(A) tail. Cap 1 was installed to improve translation efficiency. The following combinations of nucleotides were used: all unmodified nucleotides; unmodified adenosine, cytidine, and guanosine with pseudouridine (Ψ), $N^1$-methyl-pseudouridine ($m^1\Psi$), or 5-methoxy-uridine ($mo^5U$); or unmodified adenosine and guanosine with pseudouridine and 5-methyl-cytidine ($\Psi/m^5C$). After purification, the mRNA was buffer exchanged into sodium citrate buffer and stored at -20 °C until use.

## mRNA Transfection:

HeLa, Vero, BJ, AML12 and Primary Hepatocytes were seeded in 100uL per well of 96 well plates at a concentration of $2x10^5$ cells/mL one day prior to transfection and incubated overnight under standard cell culture conditions. For transfection, 50ng of mRNA was lipoplexed with 0.5uL Lipofectamine-2000 (ThermoFisher cat. no 11668027), brought to a volume of 20uL with Opti-MEM (ThermoFisher cat. no.

31985062) and directly added to cell media. All transfections were performed in duplicate.

Expression Assays:

Single endpoint Luc expression assays were conducted 24 hours post transfection, unless otherwise specified. The Luc Assay System (Promega cat. no. E1501) was used as per manufacturer's suggested protocol with 100uL lysis buffer at 1:10 dilution with Luc assay reagent. Luminescence was measured on a Synergy H1 plate reader.

Single endpoint hEpo expression assays were conducted 24 hours post transfection, unless otherwise specified. The Human Erythropoietin Platinum ELISA kit (Affymetrix cat. no. BMS2035) was used per the manufacturer's suggested protocol.

Single-endpoint eGFP expression assays were conducted 24 hours post transfection, unless otherwise specified. Fluorescence was measured at an excitation wavelength of 488nm and emission wavelength of 509nm on a Synergy H1 plate reader.

Single endpoint interferon-beta (IFN-β) expression assays were conducted on cell supernatant 48 hours post transfection. The Human IFN-Beta ELISA kit (R&D Systems cat. no. 41410) was used as per manufacturer's suggested protocol.

Luc mRNAs with $m^1\Psi$, $mo^5U$, and a negative control mRNA lacking a poly(A) tail were electroporated into AML12 cells and both protein expression and RNA abundance was assayed at 1, 2, 3, 5, 18, and 24 hours. Luciferase expression was also determined for electroporated RNA at every hour from 1 to 6 hours post electroporation in order to ensure that delivery did not dramatically change the expression phenotype.

In vivo studies:

We measured reporter protein expression from exogenous mRNA in CD-1 and BALB/C mouse models.

Lipid nanoparticle formulation of mRNA was performed through ethanol drop nanoprecipitation by mixing acidified RNA and lipids dissolved in ethanol at a 3:1 ratio (aqueous:ethanol) at a lipid molar ratio of 50:10:38.5:1.5 (ionizable : fusogenic : structural : PEG). After pH adjustment, the mRNA-loaded lipid nanoparticles were buffer exchanged into 1x PBS and stored at 4 °C until use. Final particle size and encapsulation were <100nm and >80%, respectively, with endotoxin below 10 EU/mL.

Luc mRNAs were formulated in lipid nanoparticles at a concentration of 0.03mg/mL, administered intravenously to CD-1 mice at a dose of 0.15mg/kg of body mass and measured for expression by whole body Bioluminescence Imaging (BLI) at 6 hours post injection.

hEpo mRNAs were formulated in lipid nanoparticles at a concentration of 0.01mg/mL, administered intravenously to BALB/C mice at a dose of (0.05mg/kg of body weight and measured for serum hEpo concentration using Human Erythropoietin Quantikine IVD ELISA kit (R&D Systems cat. no. DEP00) at 6 hours post injection.

UV Melting

Absorbance was measured at 260nm on the Cary100 UV Vis Spectrometer as RNA, in 2mM Sodium citrate buffer (pH=6.5), was heated from 25˚C to 80˚C at a rate of

1˚C/minute, and then cooled from 80˚C to 25˚C at a rate of 1˚C/minute. This cycle was repeated three times in total. First derivative of absorbance values were then analyzed as a function of temperature.

SHAPE-MaP

All purified mRNAs were denatured at 80˚C for 3 minutes prior to analysis. After denaturation, RNAs were folded in 100mM HEPES, pH 8.0, 100mM NaCl and 10mM $MgCl_2$ for 15 minutes at 37˚C. All RNAs were then selectively modified with 10mM 1-methyl-6-nitroisatoic anhydride (1M6) (Sigma-Aldrich cat no. S888079-250MG) for 5 minutes at 37˚C. Background (no SHAPE reagent) and denatured (SHAPE modified fully denatured RNA) controls were prepared in parallel.

After SHAPE modification, RNA was purified and fragmented using 15mM $MgCl_2$ at 94˚C for 4 minutes. Purified fragments were then randomly primed with $N_9$ primer at 70˚C for 5 minutes. Primer extension was carried out in 50mM Tris-HCl, pH 8.0, 75mM KCl, 1mM dNTPs, 5mM DTT and 6.25mM $MnCl_2$ with Superscript II reverse transcriptase (ThermoFisher cat. no. 10864014) for 3 hours at 45˚C. RNA-seq library prep was done with the NEBNext Ultra Directional RNA Library Prep Kit for Illumina (New England Biolabs cat. no. E7420) per the manufacturer's standard protocol.

RNA-seq libraries were sequenced on the Illumina MiSeq. Raw sequencing data was analyzed using the publicly available ShapeMapper software (3). The resulting reactivity data were analyzed using a sliding window (median SHAPE) approach to quantify the degree of structure at each position in the RNA, as has previously been described (4).

**Quantification and Statistical Analysis.**

Comparison of codon effects on translation

Luc expression values from 39 Luc variants were used in 865 pairwise comparisons between synonymous codons to yield p-value testing whether inclusion of specific codons impacted protein expression by ANOVA. Graph Pad software was used to determine p-values and p-values $< 0.05$ were considered significant.

Determination of Nearest-Neighbor Thermodynamic Parameters

UV-melting experiments were performed on 39 synthetic RNA duplexes with Ψ, $m^1\Psi$, and $mo^5U$ instead of uridine. The duplex sequences were designed to enable the full thermodynamic parameters for the nearest neighbor free energy contributions for each modified nucleotide to be determined.

Raw data were collected through absorbance versus duplex melting temperature profiles over six different synthetic oligonucleotide concentrations in 1M NaCl, 10mM $Na_2HPO_4$, and 0.5mM $Na_2EDTA$, pH 6.98 salt buffer. These data were then processed using *Meltwin* v.3.5 to obtain a full thermodynamic parameter set through two different methods, those methods being the LnCt/4 vs. $Tm^{-1}$ method and the Marquardt non-linear curve fit method.

Determination of structure function relationship in SHAPE data

The log normalized values for sliding window average of SHAPE reactivites from every position within the RNA were compared to the expression levels determined in HeLa cells. Linear regression was used to determine the degree of correlation between SHAPE and protein expression. A bootstrapping approach was used to determine the limits of statistical significance at each position.

Computational modeling of eGFP expression data

Time-course data was collected from HeLa and AML12 cells transfected with the computationally designed eGFP-degron mRNAs. The eGFP-degron construct reduces protein half-life to under 1 hour, so that changes in eGFP fluorescence over time directly correlate to the kinetics of translation and mRNA decay (5). Fluorescence from the transfected cells was monitored over a 20-hour time course, and total active protein levels were calculated by integrating the area under the curve. These data were used to fit a the computational model of active protein production and degradation (Figure 6C) in which rate terms for protein maturation and degradation were held constant and the translation efficiency and rate of RNA degradation were allowed to vary to find the best fit to the experimental data. Data fitting was done in python using the Scikit learn module.

**Data availability.**

Raw sequencing files (.fastq) and processed reactivities from the SHAPE-MaP structure probing experiment are deposited into GEO under the ID codes XXXXXXXXXX.

**Contact for Reagent and Resource Sharing.**

Further information and requests for resources and reagents should be directed to and will be fulfilled by Melissa J. Moore, Melissa.Moore@Modernatx.com

**Supplemental Figure/Table Legends:**

**Figure S1. Inclusion of modified nucleotides in mRNA alters protein expression, related to Figure 1.**

(A) Correlation in HeLa cells between the GC% (x-axis) and eGFP protein expression as measured by fluorescence (y-axis) for mRNA containing uridine. Values are the same as in Figure 1B

(B) Correlation in HeLa cells between the GC% (x-axis) and hEpo expression as measured by ELISA in ng/mL following transfection (y-axis) for mRNA containing uridine. Values are the same as in Figure 1C

(C) Correlations between hEPO expression as measured by ELISA in ng/mL following transfection into Hepatocytes (x-axis) vs. HeLa cells (y-axis) for 9 hEpo sequence variants containing U (left panel), $m^1\Psi$ (middle panel), and $mo^5U$ (right panel).

(D) Correlation of secreted hEpo protein production in HeLa cells (left graph) and primary mouse hepatocytes (right graph) to mean serum concentrations (y-axis) of hEpo protein in BALB-c mice following IV injection of LNP-formulated mRNA of 6 sequence variants plus one "codon optimized" variant ($E_{CO}$) (6). Data is shown for mRNA containing $m^1\Psi$ (left panel) and $mo^5U$ (right panel).

**Figure S2. Inclusion of modified nucleotides in mRNA alters Luc expression, related to Figure 2**

(A) Correlations between U% (x-axis, left column), GC% (x-axis, middle column), or codon adaptive index (CAI) (x-axis, right column) vs. Luc expression in HeLa cells (RLU) (y-axis) for 39 Luc sequence variants containing U (top row), $m^1\Psi$ (middle row), and $mo^5U$ (bottom row), with linear regressions and Pearson correlations. Values are the same as in Figure 2A.

(B) The distribution of expression levels across all variants for each nucleotide is shown as a violin plot with the median (white circle) and inter-quartile range (black lines) of expression values indicated for uridine (grey), $m^1\Psi$ (orange), and $mo^5U$ (dark purple). Distribution shown for expression levels in both AML12 cells (top panel) and primary mouse hepatocytes (bottom panel).

(C) Scatter plots of three pair-wise comparisons of Luc expression in HeLa cells for 39 Luc sequence variants for mRNA containing either U, $m^1\Psi$, or $mo^5U$. Data points representing a subset of sequence variants are labeled. Values are the same as in Figure 2A

(D) Correlation of Luc protein production in HeLa (left graphs) and AML12 (right graphs) cells vs. mean total luminescence of *in vivo* protein expression (RLU, y-axis) in CD-1 following IV injection of 1.5 mg/kg LNP-formulated mRNA for 10 Luc sequence variants containing $m^1\Psi$ (top panels) or $mo^5U$ (bottom panels).

(E) Whole body luminescence of CD-1 mice (five per group) following IV injection of 0.15 mg/kg LNP-formulated mRNA for 10 sequence variants (x-axis) containing

$m^1\Psi$ (left panel) or $mo^5U$ (right panel). Luminescence in the circled regions were used to quantify total expression shown in Figure 2C.

**Figure S3. Codon effects of inclusion of modified nucleotides on Luc expression, related to Figure 2**

(A) Grid comparisons of protein expression for 39 Luc sequence variants by global codon usage (rows) for mRNA containing uridine (left grid), $m^1\Psi$ (middle grid), or $mo^5U$ (right grid). Each row is ordered by frequency of codons in human genome with the most frequent appearing on the left. Codons for which global usage does not significantly impact protein expression relative to other codons are colored grey. Significant differences by two-way ANOVA comparisons are indicated using lines and the codon with the higher median expression value is colored green. P-values are noted by an

increasing number of asterisks for $P \leq 0.05$ (*), ≤0.01 (**), ≤0.001 (***), and ≤0.0001 (****).

(B) Expression timecourse in AML12 cells (RLU, y-axis) for 12 firefly Luc sequence variants ( x-axis) following electroporation of mRNA containing $m^1\Psi$ (left panel), or $mo^5U$ (right panel).

**Figure S4. Optical melting of hEPO mRNAs, related to Figure 3**

(A) Optical melting profiles of Luc sequence variants $L_{HS}$, $L_{82}$, and $L_{80}$ containing uridine (grey), $m^1\Psi$ (orange), or $mo^5U$ (dark purple) showing the change in UV absorbance at 260nm (y-axis) as a function of temperature (x-axis).

(B) Nearest neighbor thermodynamic parameters for Watson-crick base pairs (x-axis) for RNA containing all Ψ (yellow diamonds, values from Figure 3B), all uridine (grey circles, values from (7)), and single A-Ψ pair in context of unmodified RNA pairs (blue circles, values from (8)). The position of single modified nucleotides in each nearest neighbor for the Hudson, et al. data are highlighted in blue. In the case of AA/UU energies from Hudson, et al., energies are reported for Ψ in both the 5' (*) and 3' position of the YY dimer. Three of the seven U containing nearest neighbors contain two uridines, only one of which is replaced with pseudouridine in Hudson et al. Of the remaining four with a single uridine, two are within stated experimental error (0.15 kcal/mol) from Hudson et al. (CA/GU +0.15 kcal/mol difference and GA/CU +0.02 kcal/mol difference). There is no obvious theoretical explanation for the difference in the remaining two nearest neighbor values (CU/GA -0.67 kcal/mol difference and GU/CA -0.79 kcal/mol difference).

**Figure S5. SHAPE-MaP analysis of hEPO mRNAs containing modified nucleotides, related to Figure 4**

(A) SHAPE reactivity values for each nucleotide in hEpo sequence variant $H_AE_3$ containing $m^1Ψ$ shown as a column graph with errors. Colored columns indicate highly reactive (red), moderately reactive (grey), and lowly reactive (blue) nucleotides. The positions of the 5′ and 3′ UTRs (thin white boxes), $H_AE_3$ coding sequence (light grey box) are shown in the schematic below.

(B) Apparent total mutation rates (y-axis) for untreated (light grey, -) and treated (dark grey, +) samples of hEpo sequence variant $H_AE_3$ containing uridine (left), $m^1\Psi$ (middle), or $mo^5U$ (right) (x-axis).

(C) Apparent mutation rates only at U bases (y-axis) for untreated (light grey, -) and treated (dark grey, +) samples of hEpo sequence variant $H_AE_3$ containing uridine (left), $m^1\Psi$ (middle), or $mo^5U$ (right) (x-axis).

(D) Median SHAPE reactivity values (33-nt sliding window) for hEpo sequence variant $H_AE_3$ containing uridine (top), $m^1\Psi$ (middle), or $mo^5U$ (bottom) shown as a heatmap: highly reactive (red), moderately reactive (grey), and lowly reactive (blue). The positions of the 5′ and 3′ UTRs (thin white boxes), $H_A$ coding sequence (dark grey box), and $E_3$ coding sequence (light grey box) are shown in the schematic below.

(E) Median SHAPE reactivity values (33-nt sliding window) for hEpo sequence variants $E_{CO}$ (top) and $H_AE_3$ (bottom) containing $m^1\Psi$ (orange, left) or $mo^5U$ (purple, right) shown as a heatmap: highly reactive (red), moderately reactive (grey), and lowly reactive (blue). hEpo serum concentrations in mice from Figure 1D are shown to the right.

**Figure S6. SHAPE-MaP directed secondary structure models of hEPO mRNAs containing modified nucleotides, related to Figure 4**

(A) SHAPE-directed Minimum Free Energy (MFE) secondary structure predictions for hEpo sequence variant $H_AE_3$ containing uridine (grey, left), $m^1\Psi$ (orange, center), and $mo^5U$ (purple, right). The location of the 5′ end of the mRNA is indicated.

(B) Venn diagram indicating the number of common and unique base pairs for each SHAPE-directed MFE structures shown in E.

**Figure S7. Positional correlations of SHAPE-MaP reactivities and protein expression for hEPO mRNA. Related to Figure 4.**

Pearson correlations for SHAPE vs protein expression (y-axis) across nucleotide positions (x-axis) for 8 hEPO sequence variants containing U (top), $m^1\Psi$ (middle) or $mo^5U$ (bottom). Values are shown for protein expression in HeLa (blue) and primary Hepatacytes (green).

**Figure S8. RNA Structure and codon usage combine to determine protein expression, related to Figure 4**

(A) Median SHAPE reactivity values (y-axis, 33-nt sliding window) for firefly Luciferase sequences containing $m^1\Psi$ (top: $LF_{18/27}$ dark green and $L_{27}$ peach, bottom: $L_{18/7}$ light green and $L_7$ red) versus nucleotide position (x-axis) for the first 100 nucleotides. The positions of the 5′ UTR (thin white box), the beginning of the CDS (colored boxes) are shown. Lines indicate the position of the luciferase region swapped in the chimeric constructs (Region A)

(B) Top: schematic of 2 chimeric constructs ($LF_{18/27}$ top and $LF_{18/7}$ bottom) which combine 77 nt regions near the start codon with the remainder of the CDS of different firefly Luciferase sequence variants. Bottom: expression in primary mouse hepatocytes (RLU, x-axis) for original ($L_7$ and $L_{27}$) and fusion ($LF_{18/27}$ and $LF_{18/7}$) firefly Luciferase constructs (y-axis) containing $m^1\Psi$.

(C) Expression in HeLa cells (RLU, y-axis) for firefly Luc sequence variants from Figure 2A ($L_{18}$, $L_{CO}$) or engineered to have more stable secondary structure ($L_{HS}$) containing $m^1\Psi$ (orange) and $mo^5U$ (dark purple).

(D) Computational basepairing probabilities for the first 70 nucleotides for the secondary structure ensemble for luciferase mRNAs $L_{CO}$, $L_{18}$, and $L_{HS}$ containing $m^1\Psi$ above a schematic indicating the position of the start codon (green box). Pairing probabilities of each nucleotide is indicated by color: > 90% (red), 50 – 90 % (green), 10 – 50 % (blue), and < 10% (yellow).

**Figure S9. SHAPE-MaP analysis of computationally designed eGFP mRNAs, related to Figure 5.**

(A) Median SHAPE reactivity values (33-nt sliding window) for 6 computationally designed eGFP sequence variants shown as a heatmap below arc-diagrams of pairing probabilities from the SHAPE-directed secondary structure ensemble predictions. A schematic of the mRNA is shown below.

(B) Scatterplot of the computationally predicted minimum free energy of folding vs the folding energy SHAPE-directed secondary structure ensemble for the six mRNAs shown in Figure S9A.

**Figure S10. Positional Correlations of nucleotide composition and protein expression for computationally designed eGFP mRNAs. Related to Figure 5.**

Pearson correlations for percent U (red) and A (blue) vs protein expression in HeLa cells (y-axis) across 30 nucleotide regions (x-axis) for computationally designed eGFP sequence variants. Values are the same as in Figure 6B.

**Figure S11. Expression of designed eGFP mRNAs, related to Figure 5.**

(A) Total integrated eGFP fluorescence measured every 2 hours for 86 hours in AML12 cells (RFU, y-axis) for six sets of five mRNAs containing $m^1\Psi$ (dots, with median as black line) with differing degrees of codon optimality and/or secondary structure (x-axis, as in A).

(B) An alternative model of eGFP expression kinetics. Simulated curves based on equations for changes in levels of mRNA (mRNA, orange), immature non-fluorescent protein (inactive protein, grey), and mature fluorescent protein (fluor, green) over time using exponential decay rates for mRNA ($\lambda_{RNA}$) and eGFP protein ($\lambda_{Fluor}$), and rates of mRNA delivery ($k_{Delivery}$), translation ($k_{Trans}$), and protein maturation ($k_{MAT}$). mRNA half-lives ($t_{1/2\ RNA}$) were calculated from the observed mRNA decay rates.

(C) Correlation between the computationally determined eGFP-degron mRNA half-lives determined by computational models that excluded (Figure 6C) or include (Figure S11B) a term for delivery of the RNA.

**Supporting Table S1. Nearest neighbor base pairing energies for modified nucleotides, related to Figure 3**

Nearest-neighbor thermodynamic parameters along with the experimentally determined error for Watson-crick base pairs containing unmodified uridine (values from (7)), Ψ,

$m^1\Psi$, or $mo^5U$. The modified nucleotide(s) for each nearest neighbor pair is highlighted in red. Parameters were derived by linear regression of UV-melting data from X short oligonucleotides containing global substitutions, as described in (7).

**Supporting Table S2. Table of calculated translation efficiencies and mRNA half-lives for computationally designed eGFP variants, related to Figure 5.**

Values calculated from the model of the eGFP expression data from Figure 6. Values include, computational predicted minimum free energy of folding (MFE), Relative Synonymous Codon Usage (RSCU), Expression as calculated as area under the curve (Expr), translation efficiency ($k_{Trans}$) , mRNA half-life ($t_{1/2}$) , and model residuals.

**Dataset 1: SHAPE-MaP reactivities, related to Figure 4.**

SHAPE-MaP reactivity data for Luc and hEpo mRNAs containing uridine, $m^1\Psi$ or $mo^5U$ indicated as (U, 1-m-pU, and 5mo-U) in Row 1. The value '-999' is used to note positions of 'NO DATA' where low read-depth or high background mutation interfere with accurate quantification of SHAPE reactivities.

**Dataset 2. Table of mRNA sequences, related to Figure 1.**

A table file with the names and complete sequences for all mRNA variants of hEpo, eGFP, and Luc used in this manuscript.

**Table S1: Nearest neighbor base pairing energies for modified nucleotides**

| **Parameter** | **Uridine** (7) | **$m^1\Psi$** | **$mo^5U$** | **$\Psi$** |
|---|---|---|---|---|
| AA/UU | -0.93 $\pm$ 0.03 | -1.18 $\pm$ 0.4 | -0.64 $\pm$ 0.07 | -1.23 $\pm$ 0.05 |
| AU/UA | -1.1 $\pm$ 0.08 | -1.13 $\pm$ 0.12 | -0.77 $\pm$ 0.09 | -1.52 $\pm$ 0.14 |
| UA/AU | -1.33 $\pm$ 0.09 | -1.86 $\pm$ 0.15 | -0.95 $\pm$ 0.11 | -1.71 $\pm$ 0.16 |
| CU/GA | -2.08 $\pm$ 0.06 | -1.83 $\pm$ 0.09 | -1.60 $\pm$ 0.07 | -2.10 $\pm$ 0.10 |
| CA/GU | -2.11 $\pm$ 0.07 | -2.26 $\pm$ 0.07 | -1.87 $\pm$ 0.06 | -2.35 $\pm$ 0.08 |
| GU/CA | -2.24 $\pm$ 0.06 | -2.43 $\pm$ 0.08 | -2.00 $\pm$ 0.06 | -2.50 $\pm$ 0.08 |
| GA/CU | -2.35 $\pm$ 0.06 | -2.67 $\pm$ 0.08 | -2.30 $\pm$ 0.07 | -2.51 $\pm$ 0.10 |

## Table S2: Computational modeling of eGFP expression data

| Name | MFE | RSCU | Expr | $k_{trans}$ | $t_{1/2}$ | Residuals |
|---|---|---|---|---|---|---|
| High_RSCU_high_struc_1 | -430.8 | 0.97898762 | 62490781.9 | 0.21793898 | 7.81482687 | 0.096 |
| High_RSCU_high_struc_2 | -431.6 | 0.98134036 | 75479965.1 | 0.23048038 | 8.78031572 | 0.105 |
| High_RSCU_high_struc_3 | -429.3 | 0.97091643 | 56769972.4 | 0.18132505 | 8.26309148 | 0.055 |
| High_RSCU_high_struc_4 | -430.1 | 0.98555101 | 92330496.2 | 0.24372329 | 10.2677767 | 0.15 |
| High_RSCU_high_struc_5 | -433.6 | 0.97493502 | 99454672.7 | 0.23814752 | 11.3976759 | 0.1485 |
| Mid_RSCU_high_struc_1 | -431.2 | 0.8027473 | 138681897 | 0.36384038 | 9.84902119 | 0.1405 |
| Mid_RSCU_high_struc_2 | -431.4 | 0.79153088 | 65154513.4 | 0.32031381 | 5.16849723 | 0.0375 |
| Mid_RSCU_high_struc_3 | -431.7 | 0.80822128 | 59975694.7 | 0.25668601 | 6.04232773 | 0.0705 |
| Mid_RSCU_high_struc_4 | -432.9 | 0.79818217 | 110885258 | 0.31618165 | 8.94323151 | 0.119 |
| Mid_RSCU_high_struc_5 | -431.6 | 0.79915823 | 47054634.6 | 0.29503552 | 3.8897541 | 0.014 |
| High_RSCU_mid_struc_1 | -364 | 0.97225729 | 65181223.4 | 0.21277172 | 7.76577491 | 0.048 |
| High_RSCU_mid_struc_2 | -363.6 | 0.97009223 | 66955083.2 | 0.21511454 | 7.8763081 | 0.041 |
| High_RSCU_mid_struc_3 | -363.8 | 0.97010362 | 37722111.9 | 0.19875228 | 5.03207356 | 0.0335 |
| High_RSCU_mid_struc_4 | -363.6 | 0.97003352 | 45696217.9 | 0.24080099 | 5.05106217 | 0.0515 |
| High_RSCU_mid_struc_5 | -362.1 | 0.97056048 | 37525006.4 | 0.22034181 | 4.44846142 | 0.04 |
| Mid_RSCU_mid_struc_1 | -363.2 | 0.79800445 | 13366485.7 | 0.33145872 | 1.1091633 | 0.0075 |
| Mid_RSCU_mid_struc_2 | -362.7 | 0.79987449 | 22239789.2 | 0.22757937 | 2.59352291 | 0.0105 |
| Mid_RSCU_mid_struc_3 | -362.5 | 0.79875301 | 8735943.96 | 0.16480404 | 1.39625264 | 0.0035 |
| Mid_RSCU_mid_struc_4 | -362.2 | 0.80132337 | 17274020.7 | 0.29938124 | 1.47020018 | 0.005 |
| Mid_RSCU_mid_struc_5 | -362.3 | 0.80451192 | 998887.335 | 0.00653549 | 2.58462412 | 0 |
| Low_RSCU_low_struc_1 | -289.7 | 0.58308154 | 223554.758 | 0.00396633 | 0.69144448 | - |
| Low_RSCU_low_struc_2 | -294.2 | 0.59879943 | 35441.834 | 0.0014708 | 0.69641243 | - |
| Low_RSCU_low_struc_3 | -296.1 | 0.59967709 | 342296.988 | 0.01128865 | 0.67871313 | - |
| Low_RSCU_low_struc_4 | -297.3 | 0.59360738 | 226556.729 | 0.0066951 | 0.6863529 | - |
| Low_RSCU_low_struc_5 | -293 | 0.59755173 | 1703361.4 | 0.04481566 | 0.65460922 | - |

## Supplemental References:

1. Weinberg DE*, et al.* (2016) Improved Ribosome-Footprint and mRNA Measurements Provide Insights into Dynamics and Regulation of Yeast Translation. *Cell Rep* 14(7):1787-1799.
2. Presnyak V*, et al.* (2015) Codon optimality is a major determinant of mRNA stability. *Cell* 160(6):1111-1124.
3. Siegfried NA, Busan S, Rice GM, Nelson JA, & Weeks KM (2014) RNA motif discovery by SHAPE and mutational profiling (SHAPE-MaP). *Nat Methods* 11(9):959-965.
4. Watts JM*, et al.* (2009) Architecture and secondary structure of an entire HIV-1 RNA genome. *Nature* 460(7256):711-716.
5. Li X*, et al.* (1998) Generation of destabilized green fluorescent protein as a transcription reporter. *J Biol Chem* 273(52):34970-34975.
6. Welch M*, et al.* (2009) Design parameters to control synthetic gene expression in Escherichia coli. *PLoS One* 4(9):e7002.
7. Xia T*, et al.* (1998) Thermodynamic parameters for an expanded nearest-neighbor model for formation of RNA duplexes with Watson-Crick base pairs. *Biochemistry* 37(42):14719-14735.
8. Hudson GA, Bloomingdale RJ, & Znosko BM (2013) Thermodynamic contribution and nearest-neighbor parameters of pseudouridine-adenosine base pairs in oligoribonucleotides. *RNA* 19(11):1474-1482.

Figure S1

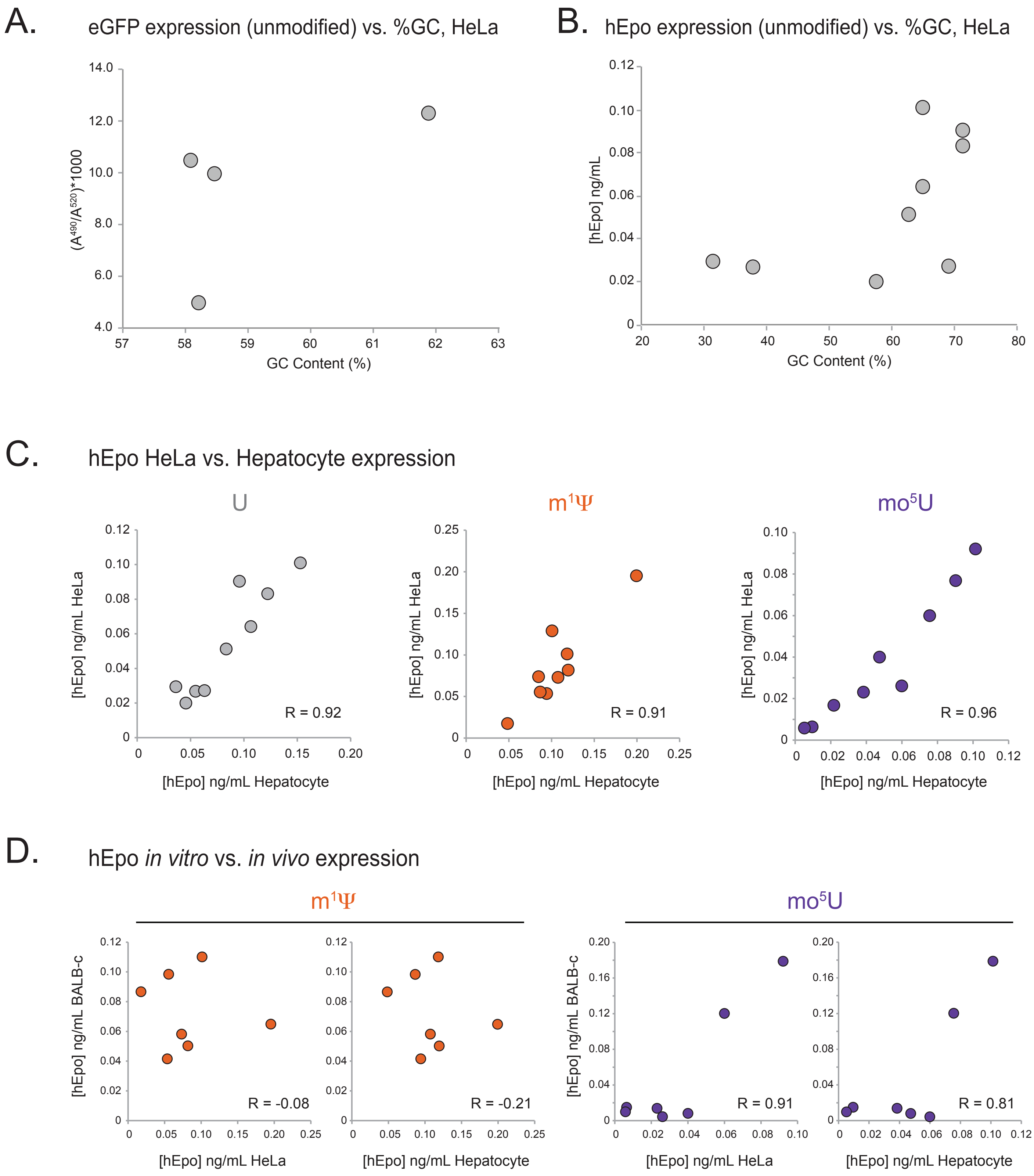
A.
eGFP expression (unmodified) vs. %GC, HeLa
14.0
12.0
10.0
8.0
6.0
4.0
(A490/A520)*1000
57
58
59
60
61
62
63
GC Content (%)
B.
hEpo expression (unmodified) vs. %GC, HeLa
0.12
0.10
0.08
0.06
0.04
0.02
0
[hEpo] ng/mL
20
30
40
50
60
70
80
GC Content (%)
C.
hEpo HeLa vs. Hepatocyte expression
U
[hEpo] ng/mL HeLa
R = 0.92
[hEpo] ng/mL Hepatocyte
m1Ψ
[hEpo] ng/mL HeLa
R = 0.91
[hEpo] ng/mL Hepatocyte
mo5U
[hEpo] ng/mL HeLa
R = 0.96
[hEpo] ng/mL Hepatocyte
D.
hEpo in vitro vs. in vivo expression
m1Ψ
[hEpo] ng/mL BALB-c
R = -0.08
[hEpo] ng/mL HeLa
R = -0.21
[hEpo] ng/mL Hepatocyte
mo5U
[hEpo] ng/mL BALB-c
R = 0.91
[hEpo] ng/mL HeLa
R = 0.81
[hEpo] ng/mL Hepatocyte

Figure S2

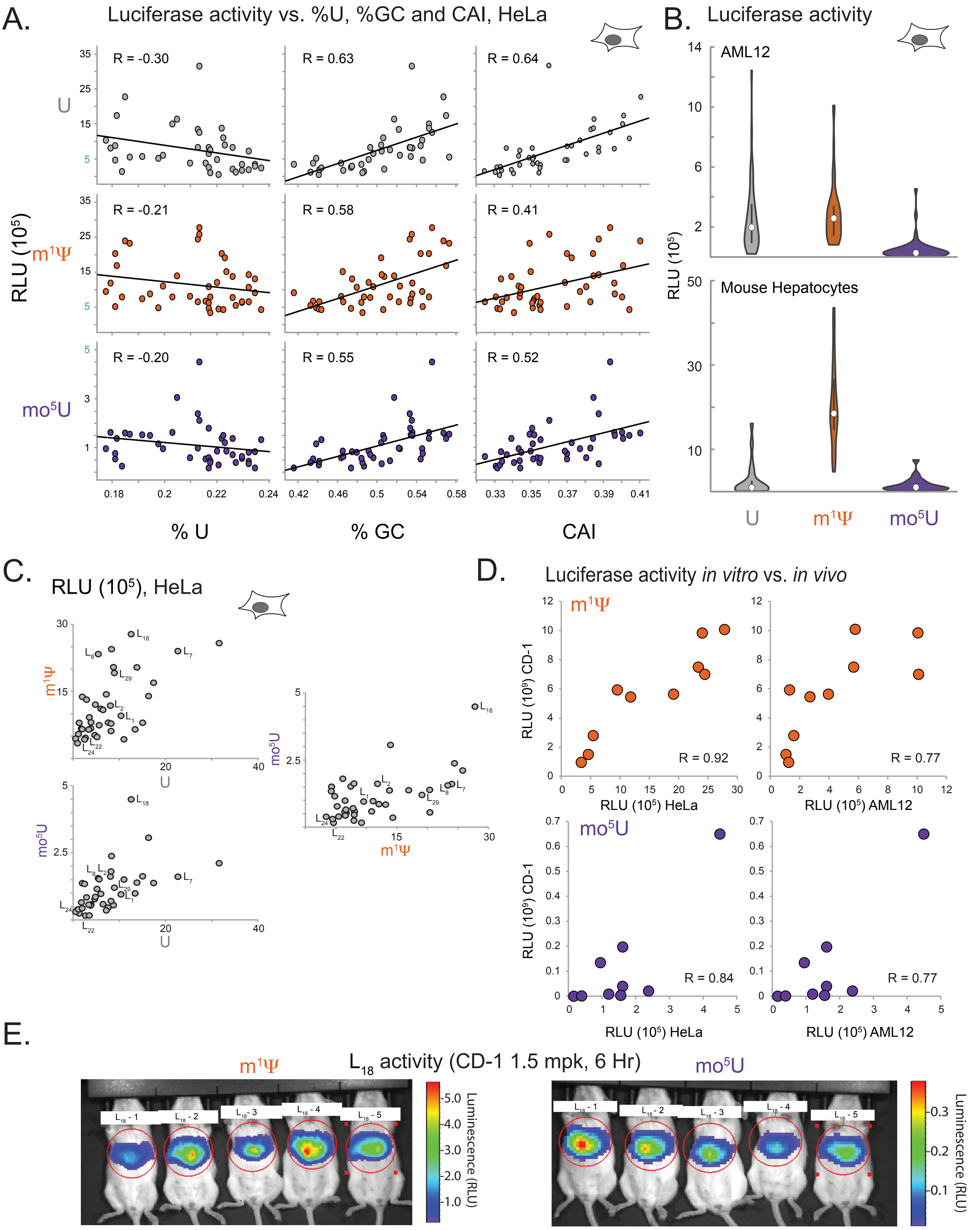
A.
Luciferase activity vs. %U, %GC and CAI, HeLa
R = -0.30
R = 0.63
R = 0.64
R = -0.21
R = 0.58
R = 0.41
R = -0.20
R = 0.55
R = 0.52
U
m1Ψ
mo5U
RLU (10^5)
% U
% GC
CAI
B.
Luciferase activity
AML12
Mouse Hepatocytes
RLU (10^5)
U
m1Ψ
mo5U
C.
RLU (10^5), HeLa
m1Ψ
mo5U
U
D.
Luciferase activity in vitro vs. in vivo
m1Ψ
mo5U
R = 0.92
R = 0.77
R = 0.84
R = 0.77
RLU (10^9) CD-1
RLU (10^5) HeLa
RLU (10^5) AML12
E.
L18 activity (CD-1 1.5 mpk, 6 Hr)
m1Ψ
mo5U
Luminescence (RLU)

# Figure S3

## A.

Luciferase activity by codon choice (pairwise comparison grids), HeLa

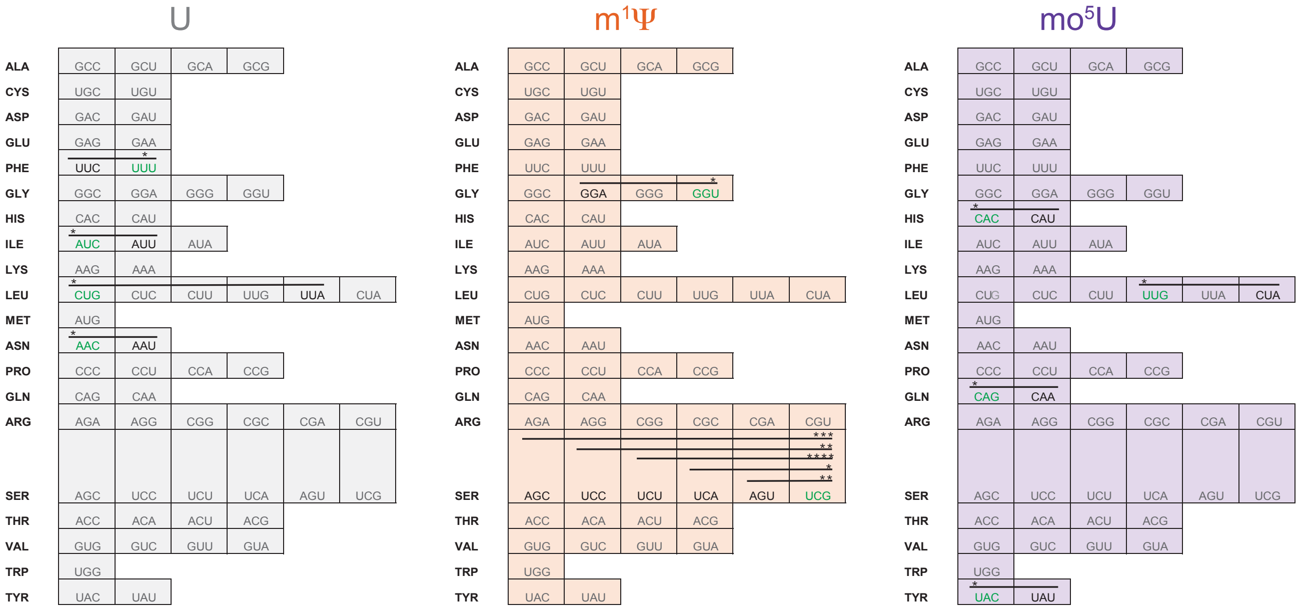


## B.

mRNA expression following electroporation

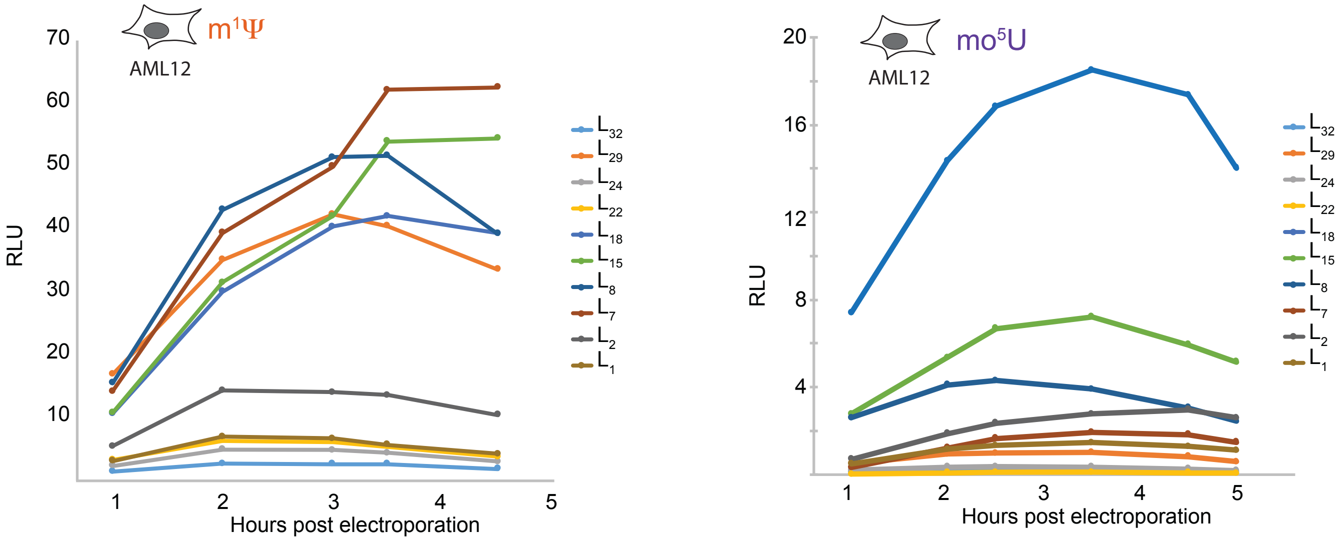

Figure S4

A.

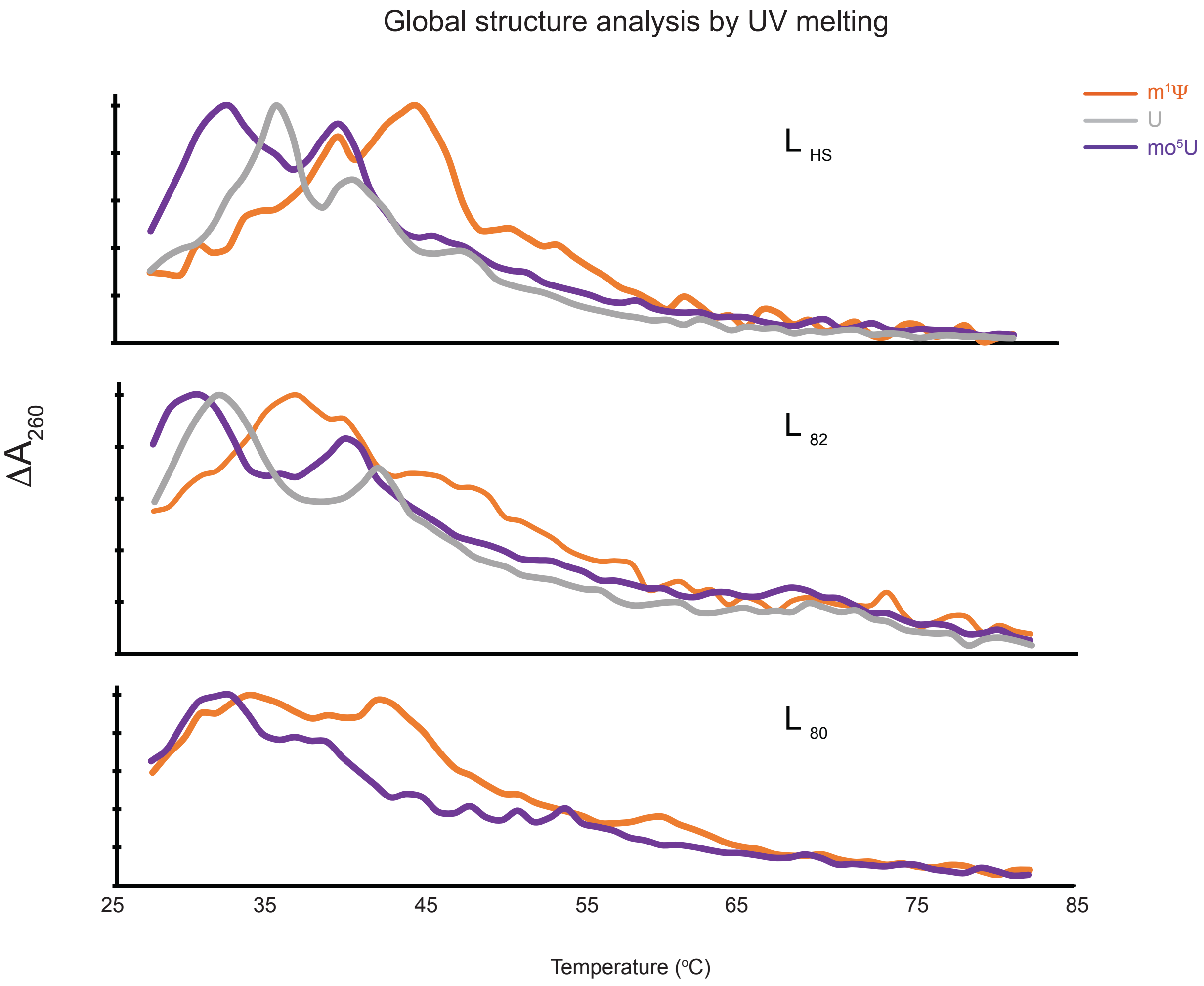
Global structure analysis by UV melting
m¹Ψ
U
mo⁵U
L HS
L 82
L 80
ΔA260
25
35
45
55
65
75
85
Temperature (°C)

B.

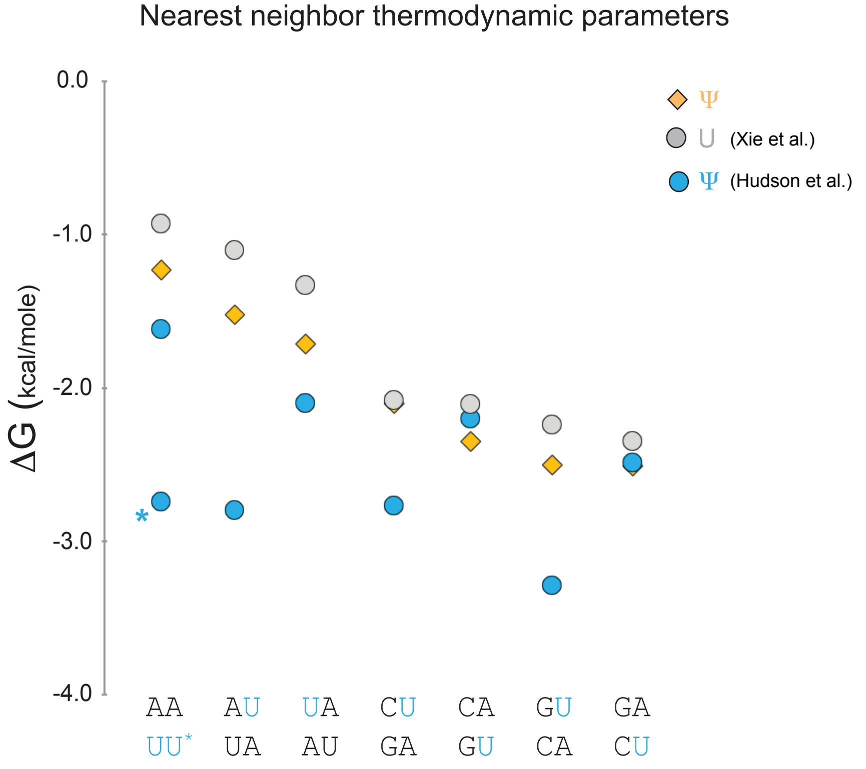
Nearest neighbor thermodynamic parameters
Ψ
U (Xie et al.)
Ψ (Hudson et al.)
ΔG (kcal/mole)
0.0
-1.0
-2.0
-3.0
-4.0
*
AA UU*
AU UA
UA AU
CU GA
CA GU
GU CA
GA CU

Figure S5

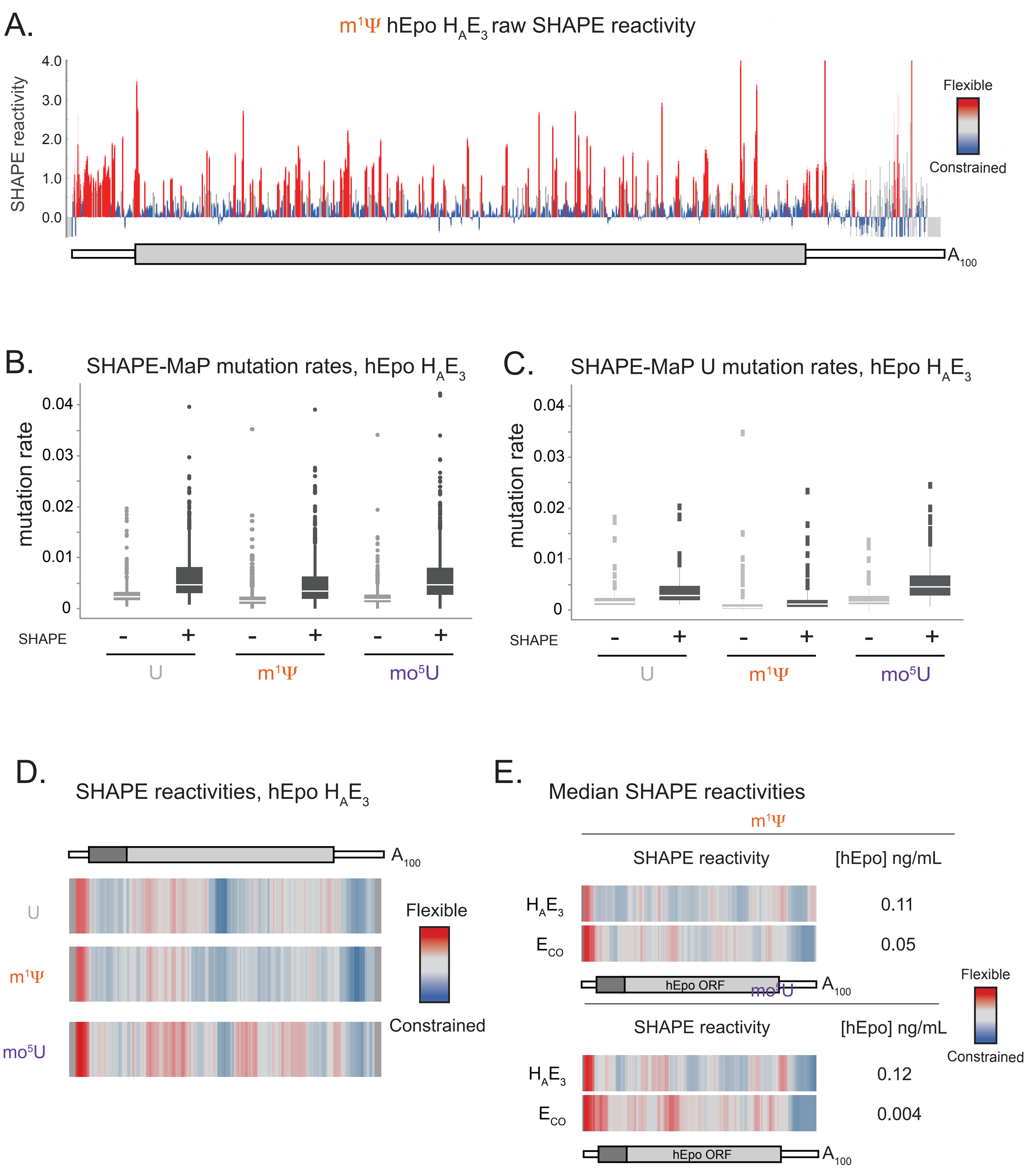

A.
m1Ψ hEpo HAE3 raw SHAPE reactivity
SHAPE reactivity
4.0
3.0
2.0
1.0
0.0
Flexible
Constrained
A100
B.
SHAPE-MaP mutation rates, hEpo HAE3
mutation rate
0.04
0.03
0.02
0.01
0
SHAPE
- + - + - +
U
m1Ψ
mo5U
C.
SHAPE-MaP U mutation rates, hEpo HAE3
mutation rate
0.04
0.03
0.02
0.01
0
SHAPE
- + - + - +
U
m1Ψ
mo5U
D.
SHAPE reactivities, hEpo HAE3
A100
U
m1Ψ
mo5U
Flexible
Constrained
E.
Median SHAPE reactivities
m1Ψ
SHAPE reactivity
[hEpo] ng/mL
HAE3
0.11
ECO
0.05
hEpo ORF
A100
mo5U
SHAPE reactivity
[hEpo] ng/mL
HAE3
0.12
ECO
0.004
hEpo ORF
A100
Flexible
Constrained

Figure S6

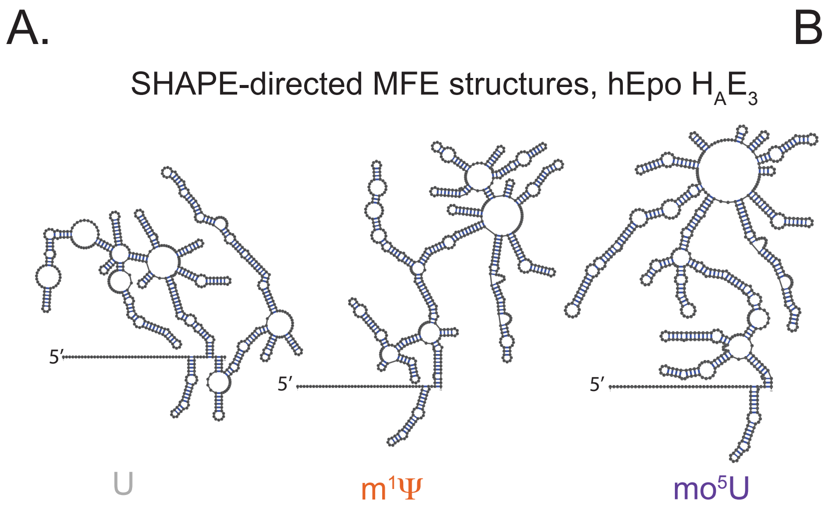
A.
SHAPE-directed MFE structures, hEpo $H_AE_3$
5′
5′
5′
U
m1Ψ
mo5U

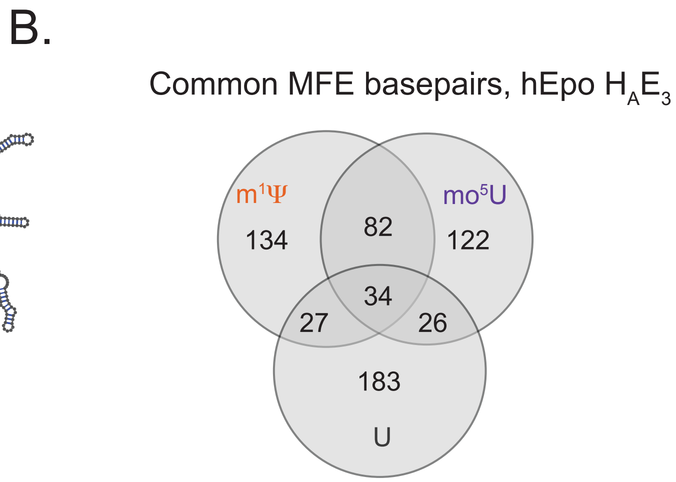
B.
Common MFE basepairs, hEpo $H_AE_3$
m1Ψ
mo5U
134
82
122
34
27
26
183
U

Figure S7

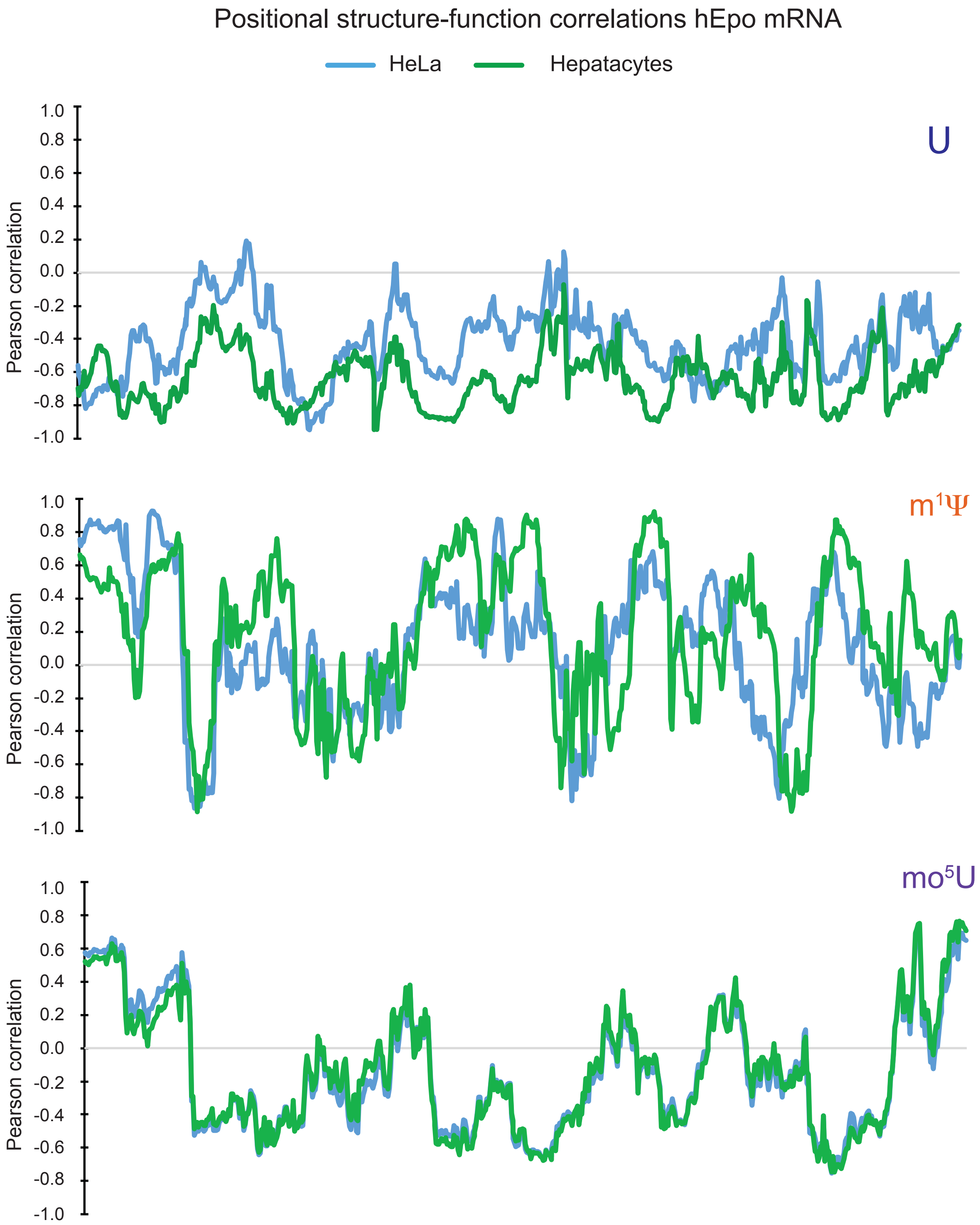

Positional structure-function correlations hEpo mRNA
HeLa
Hepatacytes
U
m¹Ψ
mo⁵U
Pearson correlation
1.0
0.8
0.6
0.4
0.2
0.0
-0.2
-0.4
-0.6
-0.8
-1.0

Figure S8

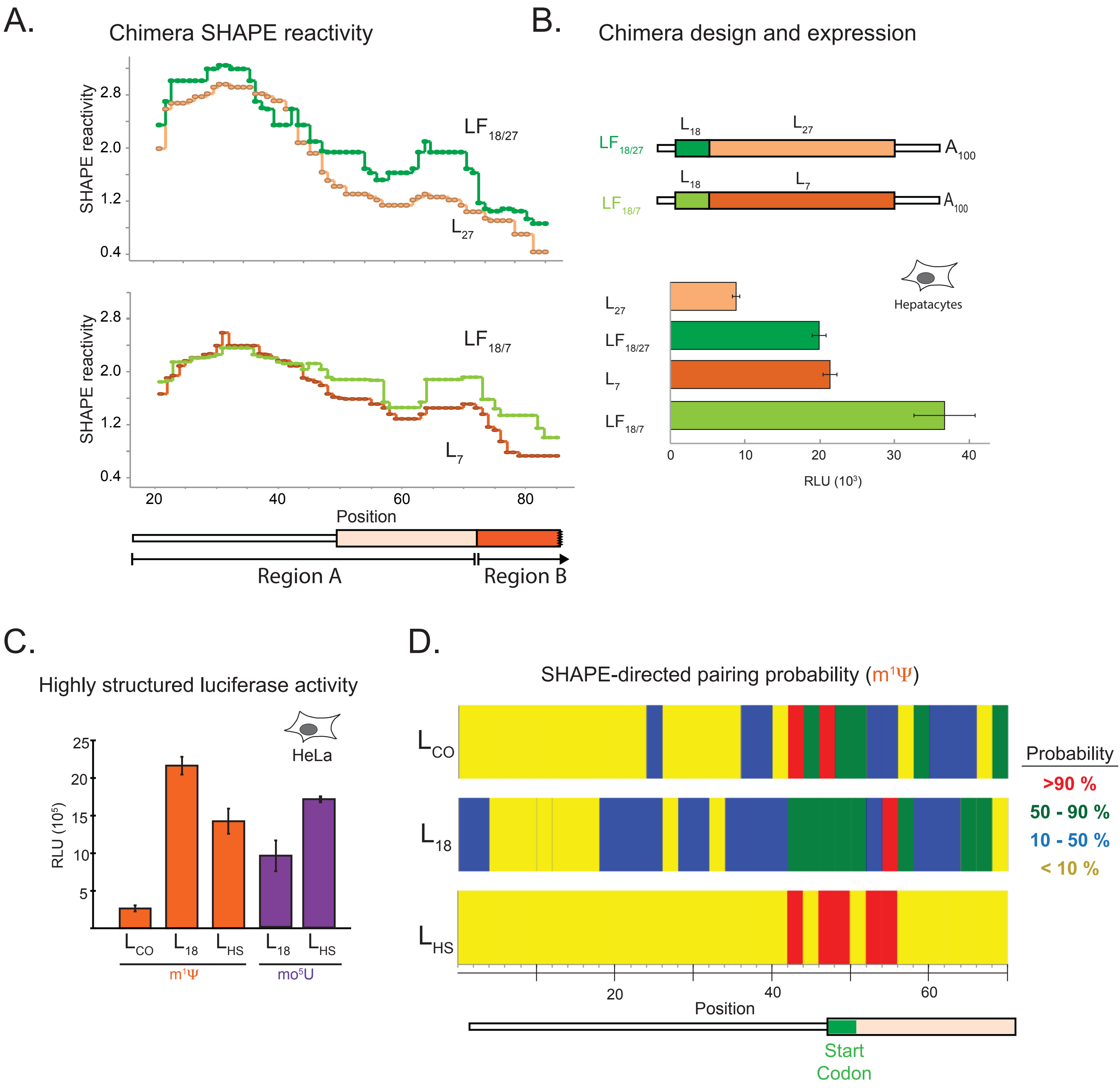

A.
Chimera SHAPE reactivity
SHAPE reactivity
2.8
2.0
1.2
0.4
LF18/27
L27
LF18/7
L7
20
40
60
80
Position
Region A
Region B
B.
Chimera design and expression
LF18/27
LF18/7
L18
L27
L7
A100
Hepatacytes
L27
LF18/27
L7
LF18/7
0
10
20
30
40
RLU (10³)
C.
Highly structured luciferase activity
HeLa
RLU (10⁵)
25
20
15
10
5
LCO
L18
LHS
L18
LHS
m¹Ψ
mo⁵U
D.
SHAPE-directed pairing probability (m¹Ψ)
LCO
L18
LHS
Probability
>90 %
50 - 90 %
10 - 50 %
< 10 %
20
40
60
Position
Start
Codon

# Figure S9

A.

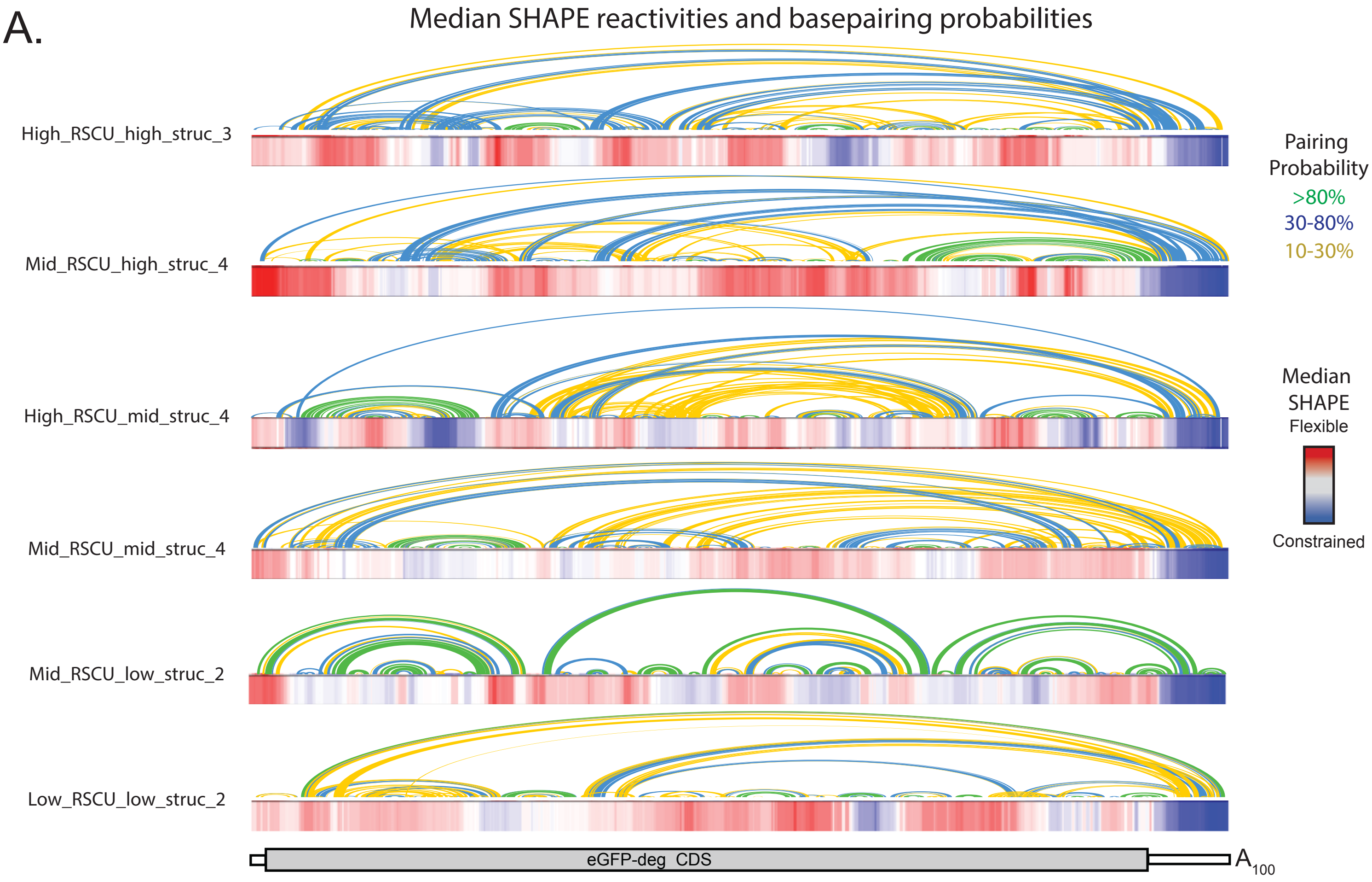
Median SHAPE reactivities and basepairing probabilities
High_RSCU_high_struc_3
Mid_RSCU_high_struc_4
High_RSCU_mid_struc_4
Mid_RSCU_mid_struc_4
Mid_RSCU_low_struc_2
Low_RSCU_low_struc_2
eGFP-deg CDS
A100
Pairing Probability
>80%
30-80%
10-30%
Median SHAPE
Flexible
Constrained

B.

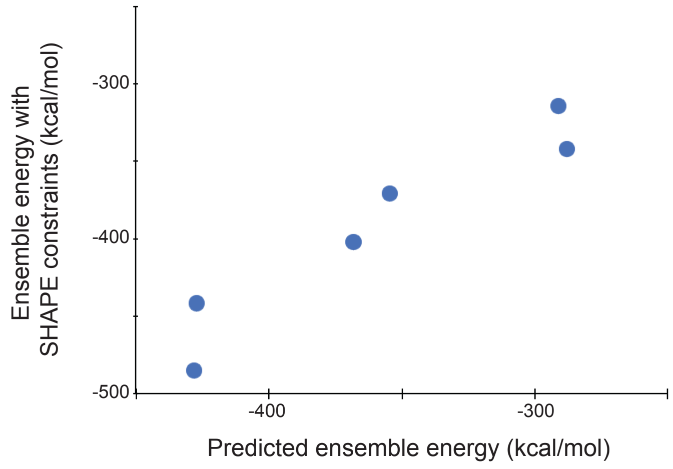
Ensemble energy with SHAPE constraints (kcal/mol)
-300
-400
-500
-400
-300
Predicted ensemble energy (kcal/mol)

# Figure S10

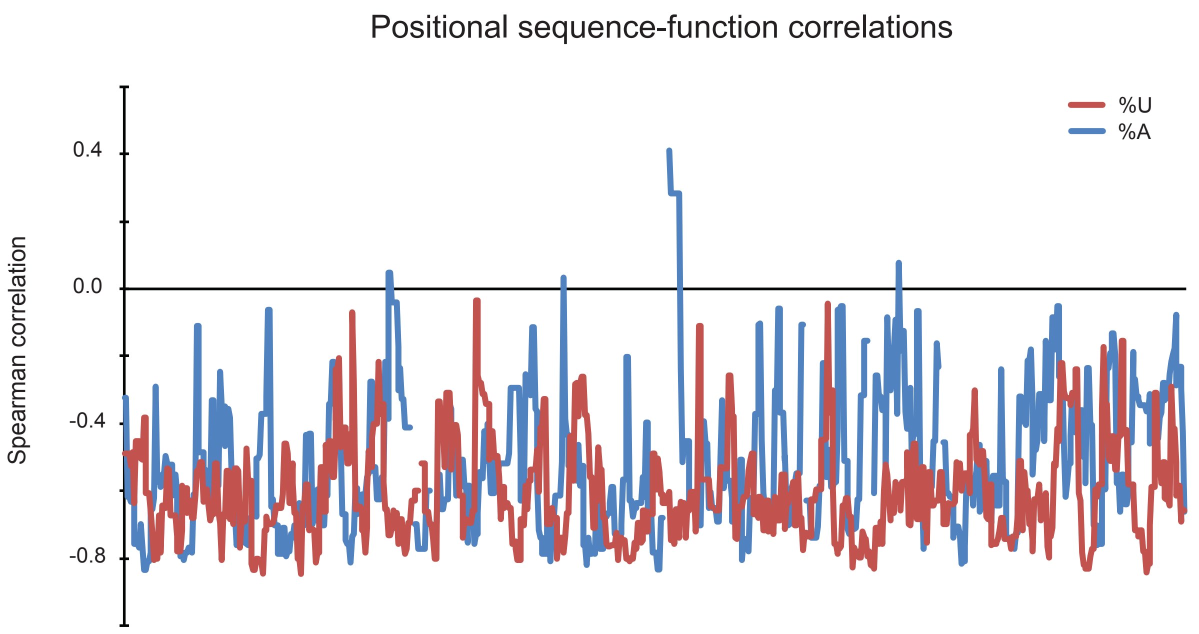
Positional sequence-function correlations
Spearman correlation
0.4
0.0
-0.4
-0.8
%U
%A

Figure S11

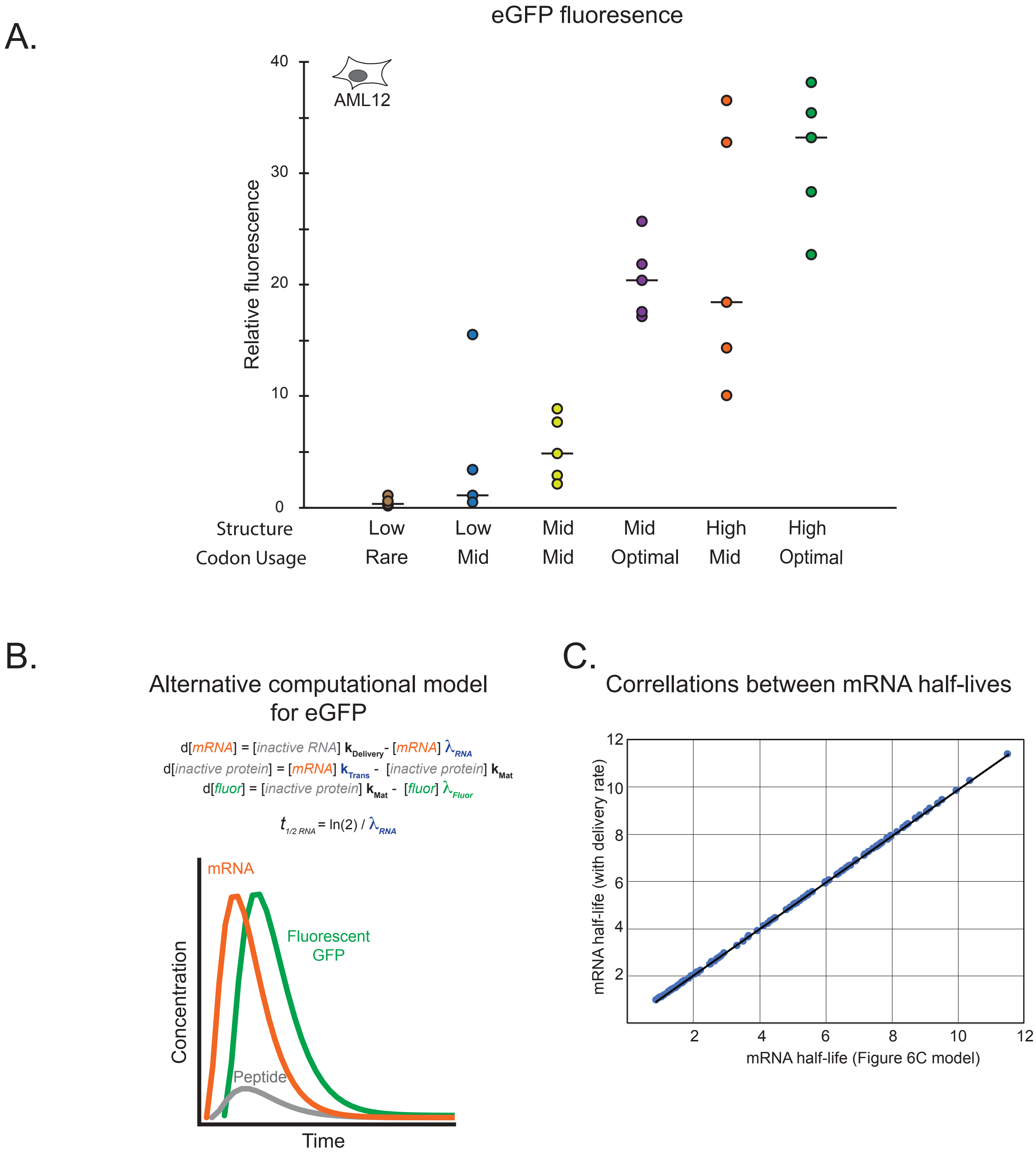
A.
eGFP fluoresence
AML12
Relative fluorescence
0
10
20
30
40
Structure
Codon Usage
Low
Rare
Low
Mid
Mid
Mid
Mid
Optimal
High
Mid
High
Optimal
B.
Alternative computational model for eGFP
d[mRNA] = [inactive RNA] k_Delivery - [mRNA] λ_RNA
d[inactive protein] = [mRNA] k_Trans - [inactive protein] k_Mat
d[fluor] = [inactive protein] k_Mat - [fluor] λ_Fluor
t_1/2 RNA = ln(2) / λ_RNA
mRNA
Fluorescent GFP
Peptide
Concentration
Time
C.
Correllations between mRNA half-lives
mRNA half-life (with delivery rate)
2
4
6
8
10
12
mRNA half-life (Figure 6C model)
